# Engineering of 2D nanomaterials to trap and kill SARS-CoV-2: a new insight from multi-microsecond atomistic simulations

**DOI:** 10.1007/s13346-021-01054-w

**Published:** 2021-09-03

**Authors:** Mohammad Khedri, Reza Maleki, Mohammad Dahri, Mohammad Moein Sadeghi, Sima Rezvantalab, Hélder A. Santos, Mohammad-Ali Shahbazi

**Affiliations:** 1 Drug Research Program, Division of Pharmaceutical Chemistry and Technology, Faculty of Pharmacy, University of Helsinki, 00014 Helsinki, Finland; 2 Computational Biology and Chemistry Group (CBCG), Universal Scientific Education and Research Network (USERN), Tehran, Iran; 3 Student Research Committee, Shiraz University of Medical Sciences, Shiraz, Iran; 4 Renewable Energies Department, Faculty of Chemical Engineering, Urmia University of Technology, 57166-419 Urmia, Iran; 5 Helsinki Institute of Life Science (HiLIFE), University of Helsinki, 00014 Helsinki, Finland; 6 Zanjan Pharmaceutical Nanotechnology Research Center (ZPNRC), Zanjan University of Medical Sciences, 45139-56184 Zanjan, Iran

**Keywords:** COVID-19, ACE2, M^pro^, SARS-CoV-2, 2D nanomaterials, Molecular dynamic

## Abstract

In late 2019, coronavirus disease 2019 (COVID-19) was caused by severe acute respiratory syndrome coronavirus 2 (SARS-CoV-2). Spike protein is one of the surface proteins of SARS-CoV-2 that is essential for its infectious function. Therefore, it received lots of attention for the preparation of antiviral drugs, vaccines, and diagnostic tools. In the current study, we use computational methods of chemistry and biology to study the interaction between spike protein and its receptor in the body, angiotensin-I-converting enzyme-2 (ACE2). Additionally, the possible interaction of two-dimensional (2D) nanomaterials, including graphene, bismuthene, phosphorene, p-doped graphene, and functionalized p-doped graphene, with spike protein is investigated. The functionalized p-doped graphene nanomaterials were found to interfere with spike protein better than the other tested nanomaterials. In addition, the interaction of the proposed nanomaterials with the main protease (M^pro^) of SARS-CoV-2 was studied. Functionalized p-doped graphene nanomaterials showed more capacity to prevent the activity of M^pro^. These 2D nanomaterials efficiently reduce the transmissibility and infectivity of SARS-CoV-2 by both the deformation of the spike protein and inhibiting the M^pro^. The results suggest the potential use of 2D nanomaterials in a variety of prophylactic approaches, such as masks or surface coatings, and would deserve further studies in the coming years.

**Graphical abstract:**

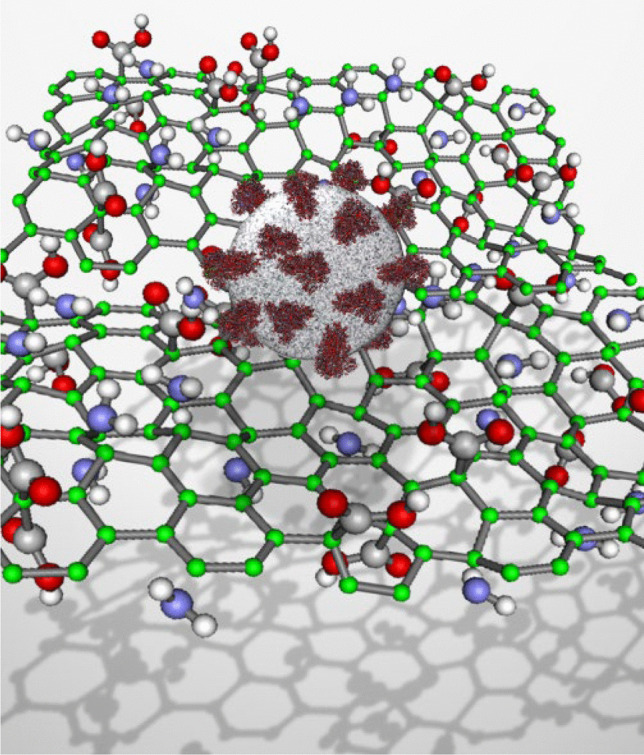

**Supplementary Information:**

The online version contains supplementary material available at 10.1007/s13346-021-01054-w.

## Introduction

With the widespread of COVID-19 pandemic, many researchers, companies, and institutes have stepped up their efforts to prevent, diagnose, and treat SARS-CoV-2 infection. Up to date, researchers have investigated the application of various nanoparticles in the detection and treatment [1–5] of SARS-CoV-2 and stimulation of the immune system [6–8]. In the last decade, the use of 2D nanomaterials (e.g., graphene and graphene oxide/dopants) have been considered in medicine due to their excellent properties and promising application in medicine, such as theranostics [9–11], antimicrobial applications [12], biosensing [13, 14], and tissue engineering [15].

**Fig. 1:**
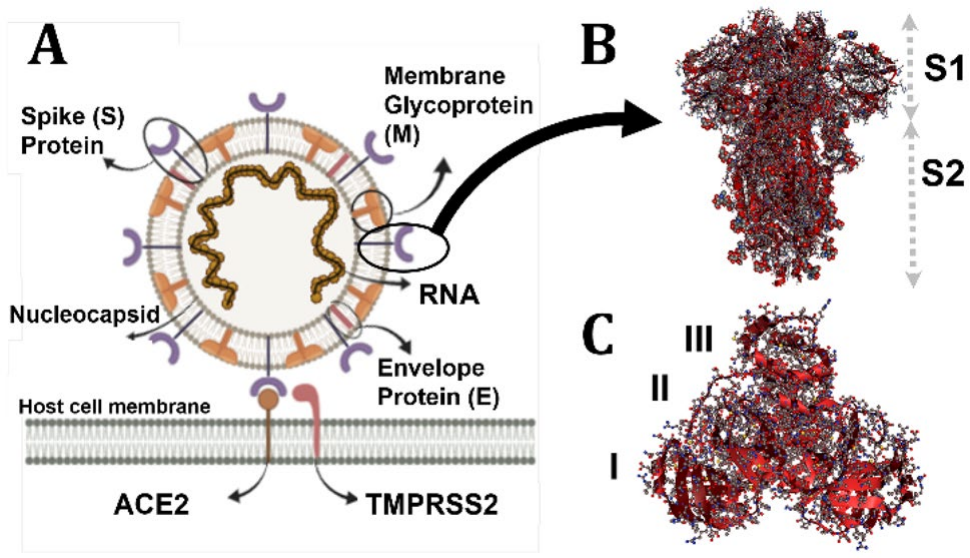
(**A**) Schematic structure of SARS-CoV-2 and the interaction of viral S protein with host cell membrane-bound ACE2. TMPRSS2 is the host cell superficial protein responsible for priming S protein after attachment to ACE2. (**B**) The subunits of **S** protein. (**C**) The structure of M^pro^ homodimer. Both monomers orthogonally aligned to each other and each monomer is composed of three main domains (domain I, II, and III)

In this regard, we hypothesize the possible application of 2D nanomaterials in the crucial battle against COVID-19. Molecular dynamic studies seem a promising approach to unravel essential information about the 2D nanomaterials’ interaction with the SARS-CoV-2 at the atomic level before further and larger-scale studies [16]. The causative agent of this disease, SARS-CoV-2, is a member of the beta-coronavirus family. SARS-CoV-2 genetic material (Fig. 1A) is composed of RNA and exists in the nucleocapsid, which is surrounded by a lipid bilayer [17]. On its outer surface, three structural proteins of the membrane (M), envelope (E), and spike (S) are anchored, with an overall diameter of 0.1 μm [18]. As a result of the large size of SARS-CoV-2, it is impossible to simulate the entire sphere of the virus at once. The solution we came up with is to consider separate parts of the virus to predict the contact of 2D nanomaterials from varying aspects and sides.

Spike protein consists of two subunits, S1 and S2 (Fig. 1B). The S1 subunit on the outer part of the protein consists of two parts, including N-terminal (S1-NTD) and C-terminal (S1-CTD). Both terminals of the S1 subunit are involved in interacting with host cell receptors and infection transmission. One of the most essential receptors that mediate the spike protein binding to host cells is angiotensin-I-converting enzyme-2 (ACE2) [19], which is widely expressed in the respiratory tract.

Researchers have studied the structure [20, 21] and the function [22] of spike protein and possible blockers [23–26] to target and suppress the virus through its spike protein. For instance, Han and Kral [27] designed peptide inhibitors with α-helix structure against spike protein. These structures were extracted from the basic structure of ACE2. They suggested that these peptides can be used by inhalation for prophylactic applications [27]. In another study, Gorshkov et al. [28] designed a novel nano-conjugation probe using quantum dots to quickly study the interactions of ACE2 and spike proteins.

The SARS-CoV-2 has a complex structure that is enclosed in an envelope membrane [29]. The lipid bilayer membrane supports the whole assembly of the virus genome and especially keeps the spike proteins in place to fuse into ACE2 [30]. Flexing and softening of the membrane can interfere with the function of other parts, such as spike proteins [30, 31]. Hence, another approach is the destabilization and even deactivation of SARS-CoV-2 through the phospholipid membrane [32]. This is an advantageous feature that sanitizers and disinfectants use to inactivate the virus [33]. Therefore, the phospholipid membrane might be targeted by 2D nanomaterials to attack the virus from all directions.

Virus docking to the human host cells happens upon attaching the spike protein to ACE2. Therefore, inhibiting or inactivating this protein will play an important role in controlling the disease. Since the transmission of the virus takes place mostly with respiratory droplets [34, 35], the first step to inactivate the virus can be taken before it enters the body. In this regard, the use of facial masks seems necessary [36] and consequently compulsory in many countries worldwide [37]. Although many types of face masks, especially surgical grades, are reported as efficient protectors from coronavirus transmission [38], many attempts have been made to improve the efficiency of filtration [39, 40]. For example, El-Atab et al. prepared hydrophobic and self-cleaning membranes to be used on the masks with a polyimide on Si-wafer. In several reports, researchers have also attempted to modify hydrophobicity [39], photothermal [41], and electrothermal [42] properties of the masks with graphene nanomaterials. In this line, due to the antiviral properties of graphene and its derivatives, several applications to combat COVID-19 have been proposed and investigated [43–46]. For example, Maio et al. [47] suggested the utilization of antiviral properties of graphene and graphene oxides on facial masks to reduce the infection capability of the SARS-CoV-2. Seo et al. [48] introduced a highly sensitive biosensor for rapid SARS-CoV-2 detection, which was produced by coating anti-spike protein antibodies onto the graphene sheets. 2D nanomaterials have shown promising properties to be utilized in biomedical applications [49]. These 2D nanomaterials include different families where graphene and its derivatives are very well-known [50, 51]. Phosphorene has also been introduced as a new class of biocompatible alternative for graphene in medical science [52, 53]. For example, Zhang et al. [53] reported better performance of graphene compared to the phosphorene in the disruption of villin headpiece (HP35) function. The less disruption by phosphorene was attributed to the weaker dispersion interactions with proteins, as a result of Lennard–Jone potential parameters and surface morphology of the phosphorene. Han et al. [54] evaluated borophene exposure to plasma proteins at varying concentrations and sizes and compared the protein corona formation at the surface of borophene with protein coronas formed at the surface of graphene and phosphorene from different perspectives like immunoregulatory features. Bismuthene is also a monoelemental 2D structure similar to graphene that is composed of a layer of bismuth element, which can be utilized as another alternative for graphene in biomedical applications [55].

Another aspect of the combat against the COVID-19 is the inhibition of its proteases since after virion capture (with spike protein and internalization), main protease (M^pro^) together with papain-like protease (PL^pro^) is responsible for the virus replication [43]. The M^pro^ is composed of three domains (Fig. 1C). Its main function is mediated by domains II and III [56]. In the case of infection, deactivation of the virus in the body can be achieved by avoiding the virus’ replication and propagation. It is notable that the M^pro^ is the best-characterized target of SARS-CoV-2 for therapeutic interventions [57]. Researchers utilized diverse methods to study the function and structure of M^pro^ [58, 59] and screen and explore various therapeutics [60, 61]. For instance, by employing molecular docking, Farag et al. [62] revealed a group of drugs (with Rosuvastatin as the best among the list) as potential therapeutics that can be considered for further in vivo and in vitro studies.

In this study, the interaction of crystalline 2D nanomaterials (bismuthene, graphene, phosphorene, P-doped graphene, and functionalized p-doped graphene) with SARS-CoV-2 is comprehensively studied. Herein, we aim to investigate the potential of 2D nanomaterials toward the deactivation of SARS-CoV-2 in facial masks and air filters. Firstly, using molecular methods based on computational analysis, we studied the potential applications of 2D nanomaterials in facial masks with the effect of 2D nanomaterials on spike protein, and afterward the interaction of deformed spike proteins with ACE2 receptors. Since the interaction between spike protein and ACE2 is a vital step in SARS-CoV-2 pathogenesis, disrupting the structure of spike protein by 2D materials is a rational way to stop the transmission of this trouble-making virus. Furthermore, the impact of the abovementioned 2D nanomaterials is investigated on the phospholipidic membrane of the virus. Ultimately, the 2D nanomaterials are brought into contact with M^pro^ of the virus to explore their therapeutic effects.

## Methodology

Bismuthene and phosphorene structures were obtained from literature references [63–66] and designed using Avogadro software [67]. Online Resource Fig. S1 shows the structural details of the 2D structures. The graphene structure was also designed by Nanotube_Modeler_1.7.9 software (http://www.jcrystal.com/products/wincnt/). Then, using Avogadro software, 50% of the carbon atoms were replaced with phosphorus atoms, and the carboxyl functional group was added to the P-doped graphene structure. Finally, the esp charge of molecular structures was calculated using the B3LYP method with base set, 6–31 + g * and CP2K software [68]. Moreover, the molecular structure of M^pro^ was extracted from complexes with Protein Data Bank (PDB) IDs 6LU7, 6W63, 7JQ1, 7JQ4, and 7JQ5. The structure of these complexes is available on the RCSB website (https://www.rcsb.org/). Also, the molecular structures of spike protein and ACE2 were downloaded from RCSB website using PDB ID 6M0J. Also, the structure of the membrane was made of dipalmitoyl phosphatidylcholine (DPPC), which was downloaded from the Charmm-GUI server of the martini website [69].

Initially, the simulation of M^pro^ and spike protein in aqueous media was performed in two independent coarse-grained simulations. Then, the changes of the M^pro^ and spike protein structure caused by 2D structures were surveyed in 10 independent simulations in aqueous media. The effect of 2D structures on the deformation of the spike protein structure was investigated using all-atom simulation. In order to investigate the sequelae of spike protein deformation, the interaction between ACE2 and spike protein deformed by each of the 2D structures was surveyed in 5 independent simulations in aqueous media. In this work, simulations were performed using GROMACS 2019.5 software [70].

OPLSA force field [71–73] was used in all-atom simulations. To perform these simulations, spike protein and 2D structures were placed in 10 × 10 × 10 nm^3^ boxes. At NVT and NPT stages, the temperature and pressure of boxes reached equilibrium at 300 K and 1 bar, using v-rescale and Parrinello_Rahman algorithms. Finally, the simulations were performed using the LINCS algorithm, with the cut-off radius of 1.5 nm, and the H-bonds were considered in 100 ns with steps of 2 fs [74]. It should be noted that DSSP analyses are provided from all-atom simulations.

Simulation of interactions between biological molecules over a long period of time requires the use of coarse-grained simulation. In this regard, the Martini force field has been used in this study. This force field has been considered by researchers in this field due to its high ability to predict intermolecular forces and, consequently, the dynamic behavior of biological molecules. The use of this force field makes it possible to accurately predict the behavior of biological molecules [75–81]. Therefore, coarse-grained simulations were performed using the Martini force field [75]. The topologies of molecular structures were created using the Python scripts available on the Martini site (http://cgmartini.nl/index.php/martini). V-rescale and Parrinello_Rahman algorithms [75] were used to equilibrate the simulation system at 300 K and 1 bar. The simulations were then performed by considering the cut-off radius of 3 nm and with steps of 20 fs at 3000 ns. Simulation boxes were designed for these systems in the size of 25 × 25 × 25 nm^3^.

In addition to molecular dynamics simulations, docking simulations were used to investigate the interaction of spike protein and ACE2. The PDB files related to deformed spike protein were obtained from all-atom simulations. In order to do this, a Gasteiger charge was added to the spike protein PDB files, and the docking process of spike protein and ACE2 was performed in autodock_vina_1_1_2_linux_x86 software [82]. To validate our method, we replicated three simulations in three previous separate works by our methodology and compared them, which confirmed our results. Detailed results of this validation are presented in the online resource Fig. S2.

## Results and discussion

### Evaluation of the effect of 2D nanomaterials on SARS-CoV-2 spike protein

**Fig. 2:**
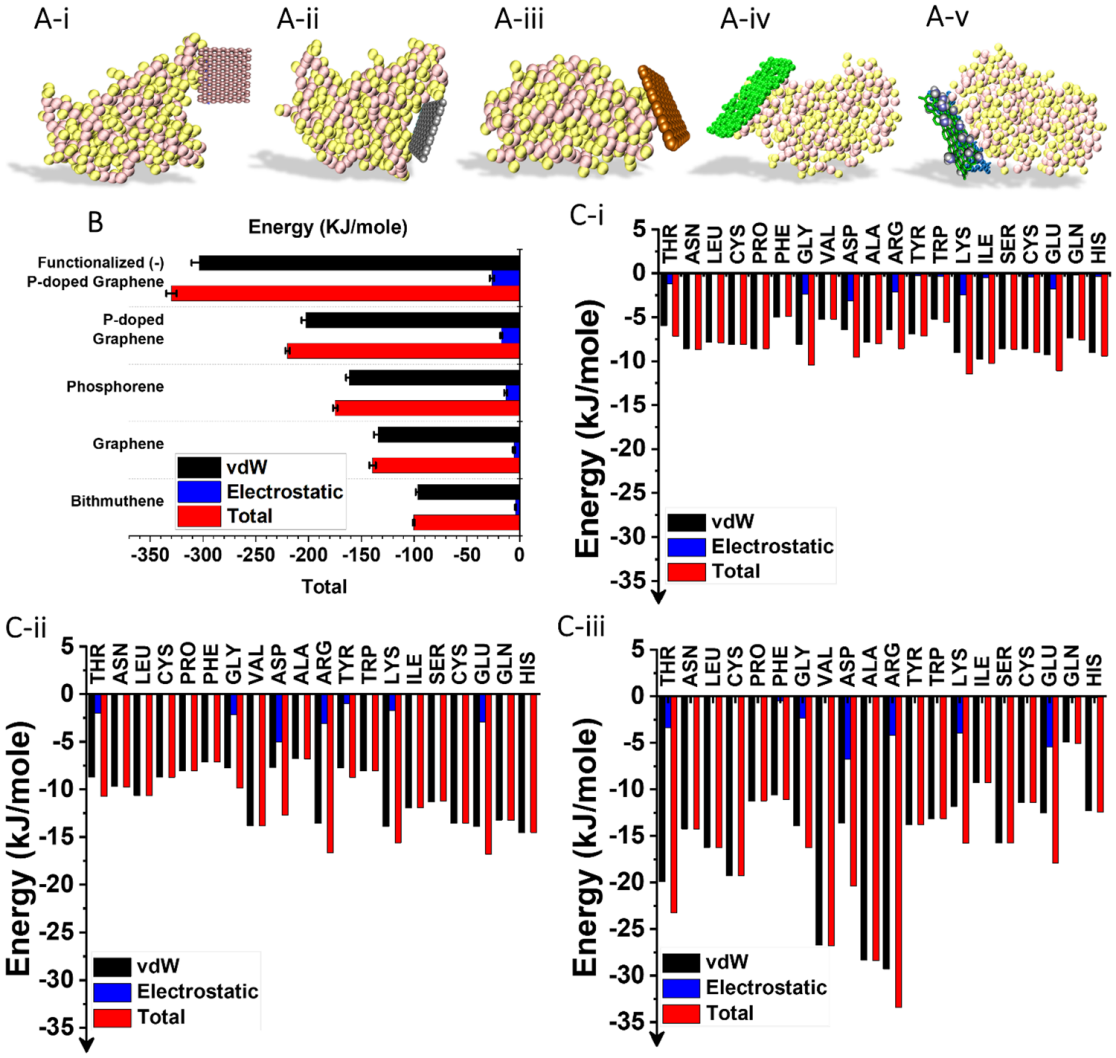
(**A**-i–v) Snapshots of spike protein interaction with bismuthene, graphene, phosphorene, P-doped graphene, and functionalized (-) P-doped graphene, respectively (Videos S1-S5). (**B**) Energetic analysis of the interaction between 2D nanomaterials and spike protein. (**C**-i–iii) Analysis of the interaction of spike protein residue with phosphorene, p-doped graphene, and functionalized (-) P-doped graphene, respectively. Snapshots of the last stage of the simulation are provided on each plot. Error bars are shown in Table S1

At the first stage of this study, the interaction of spike protein and 2D nanomaterials that results in the deformation of spike protein and change in its transmissibility was examined (Fig. 2A). The total energy of the interactions, including van der Waals (vdW) and electrostatic, is shown in Fig. 2B. It is notable that in all cases, vdW interactions have a decisive role in total energy. Surface tuning can readily improve the interaction of nanomaterials with spike protein; i.e., doping of graphene lattice and decoration of the surface with carboxylate groups boasted the electrostatic energy from −5 kJ/mole to more than −26 kJ/mole. It indicates the critical role of surface engineering. The overall interaction of spike protein is the result of the individual residues interactions. In this regard, the contribution of residues is plotted in Fig. 2C and in online resource Fig. S3. According to the results, twenty residues are involved in the binding of nanomaterials to spike protein. Shifting from graphene and phosphorene to p-doped graphene alters the residues’ contribution to the overall binding energy. For instance, histidine (HIS), glutamine (GLN), and aspartic acid (ASP) have the strongest binding with graphene (−10.35, −15.24, −12.48 kJ/mole, respectively) [83], while in the case of phosphorene, they contribute to the total energy in the scale of other residues (approximately −7.5 to 9.5 kJ/mole). The combination of phosphorous and carbon atoms to form the p-doped graphene allows the benefit from both properties in the attraction of spike protein. For example, the contribution of Serine (SER) residue for individual graphene and phosphorene nanomaterials is −3.71 kJ/mole and −8.57 kJ/mole, respectively, while the value drops to −11.25 kJ/mole for p-doped graphene.

The comparison of functionalized (-) p-doped and p-doped graphene demonstrates that the addition of carboxylate groups on the surface intensifies the interaction with negatively charged residues (e.g., threonine (THR), arginine (ARG), valine (VAL)) that adds up to the total binding energy. Moreover, this modification not only increases the electrostatic energy but also makes vdW attractions between spike protein and the nanosheet stronger. The stronger the interaction, the more the spike protein is deformed. In the following section, for each case, a long time-scale simulation (3000 ns) was performed to test the effect of nanomaterials on the deformed protein with the ACE2 receptor.

### Evaluation of the interaction of SARS-CoV-2 spike protein with ACE2 receptor

**Fig. 3:**
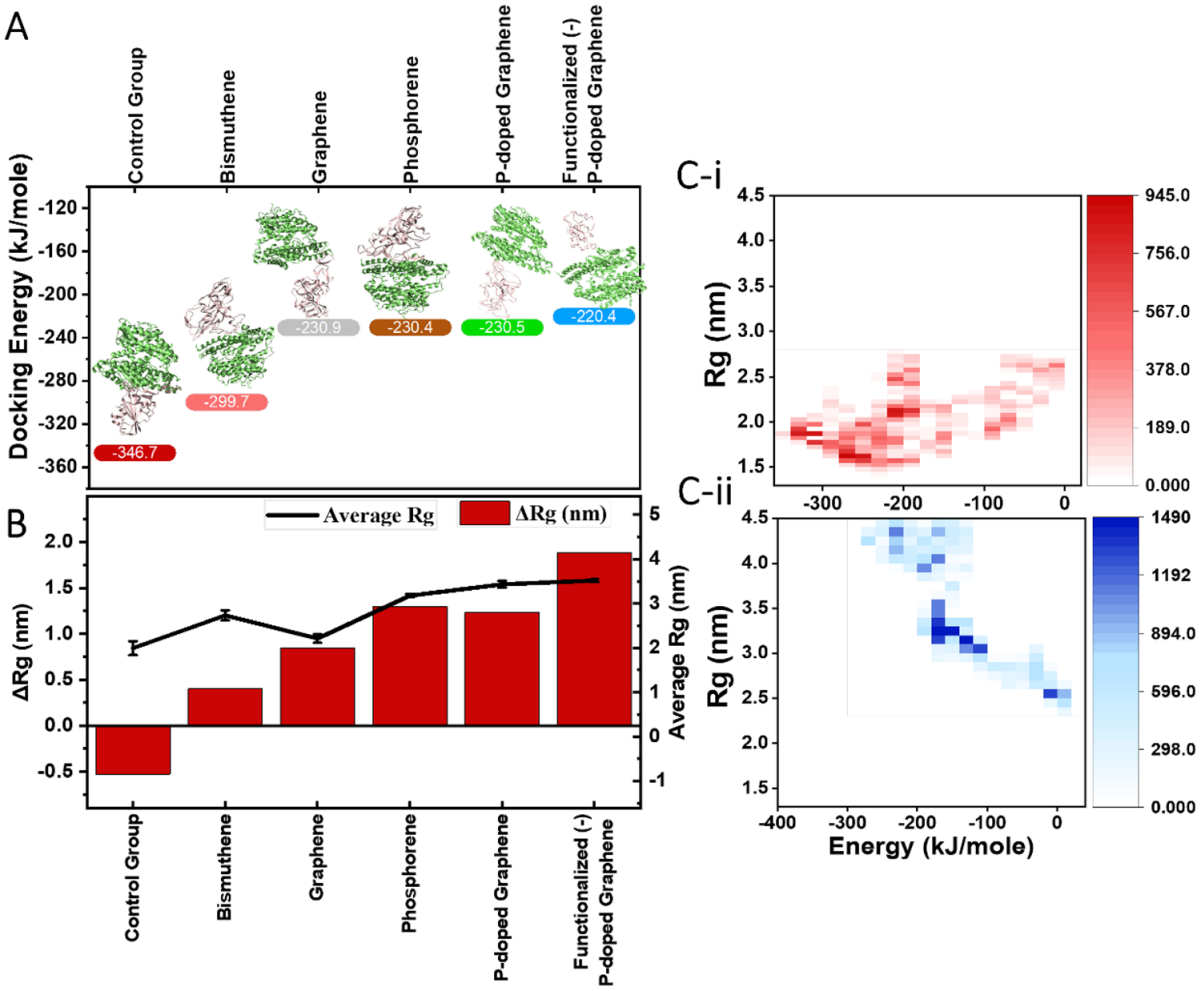
The evaluation of the interactions of spike protein with 2D nanomaterials. (**A**) Docking analysis of the binding affinity of spike protein with the ACE2 receptor. (**B**) Analysis of the radius of gyration for all samples with average Rg and the ΔRg (difference of final and initial Rg). (**C**) Mapping Rg vs. energy of binding for the (i) control group and (ii) functionalized (-) p-doped graphene

The study of structural changes in the spike protein is important for the diagnosis, treatment, and prevention of COVID-19. Therefore, an approach to analyze the nanomaterials’ impact on this protein is to evaluate the binding affinity of the deformed spike protein and ACE2. Figure 3A outlines the binding energy of pristine spike protein fragments (hereinafter called the control group) and the deformed protein fragments with different nanomaterials. As it is clear, the lowest binding energy and consequently the most stable complex of spike protein and ACE2 is related to the pristine sample with ca. −346.7 kJ/mole. Considering the interaction of spike protein deformed by phosphorene and graphene with ACE2, it is understood that the performance of all these nanomaterials is similar; however, because of the lower biodegradability in vivo and higher cytotoxicity of graphene, phosphorene has priority in the biological environments [52]. Moreover, oxygen exerts dissociating effects on the phosphorene nanomaterials [84]. Therefore, utilizing these 2D nanomaterials in the facial masks and air filters that are exposed to air can weaken their antiviral effect since it can be degraded at a higher rate compared to graphene. To take advantage of both nanomaterials, phosphor-doped graphene nanomaterials were designed and considered in the simulations to monitor its impact on SARS-CoV-2 spike protein. Interestingly, docking analysis revealed that the nanomaterials carry the advantages of both original nanomaterials and deform the spike protein in the way that its affinity toward ACE2 is not significantly changed (E ~  −230.5 kJ/mole). To improve the physicochemical properties of the resultant nanomaterial, its surface was decorated with carboxylate groups to improve the electrostatic interactions of spike protein and nanomaterial to result in more deformation of the spike protein. The binding energy of the affected spike protein with the functionalized (-) nanosheet revealed the interaction with ACE2 when compared with other spike proteins.

Structural and functional analysis of SARS-CoV-2 revealed that during the binding, spike protein’s structure transforms from prefusion to postfusion; i.e., at the later stage, it forms a long needle-like structure [6, 21]. In this regard, to further quantify the binding of spike protein (pristine and deformed) with ACE2 receptor, we examined the average radius of gyration and ΔRg (Rg_final_ − Rg_initial_) for each nanosheet (Fig. 3B). The lower the ΔRg, the more advanced stage of binding the virus can go through. As it can be seen, the tightest structure is related to the control group, where the final Rg size is lower than its initial state as indicative of postfusion structure. Spike protein distorted by functionalized (-) p-doped graphene and phosphorene 2D nanomaterials exhibits the most extended structures. The results confirm the inhibitory effects of the employed nano-objects.

MD simulation of the study cases reveals more detail on the interactions. Figure 3C and online resource Fig. S4 represent the Rg of spike protein as a function of interaction energies for spike protein with ACE2. For example, the minimum energy range locates at ca. −310 kJ/mole, 1.92 nm for the control group, while this value for the spike protein deformed with phosphorene and graphene shifts to ca. −185 kJ/mole, 3.55 nm, and ca. −210 kJ/mole, 2.1 nm, respectively. P-doping and functionalizing the graphene pushes the centers toward ca. −150 kJ/mole, 3.8 nm, and ca. −130 kJ/mole, 3.2 nm, respectively. It is obvious that surface engineering of the 2D nanomaterials provides higher energetic interaction (i.e., less stable) with ACE2 and the higher extended structure of spike protein. Altogether, these effects avoid spike protein to form a stable complex with ACE2 receptors.

**Fig. 4:**
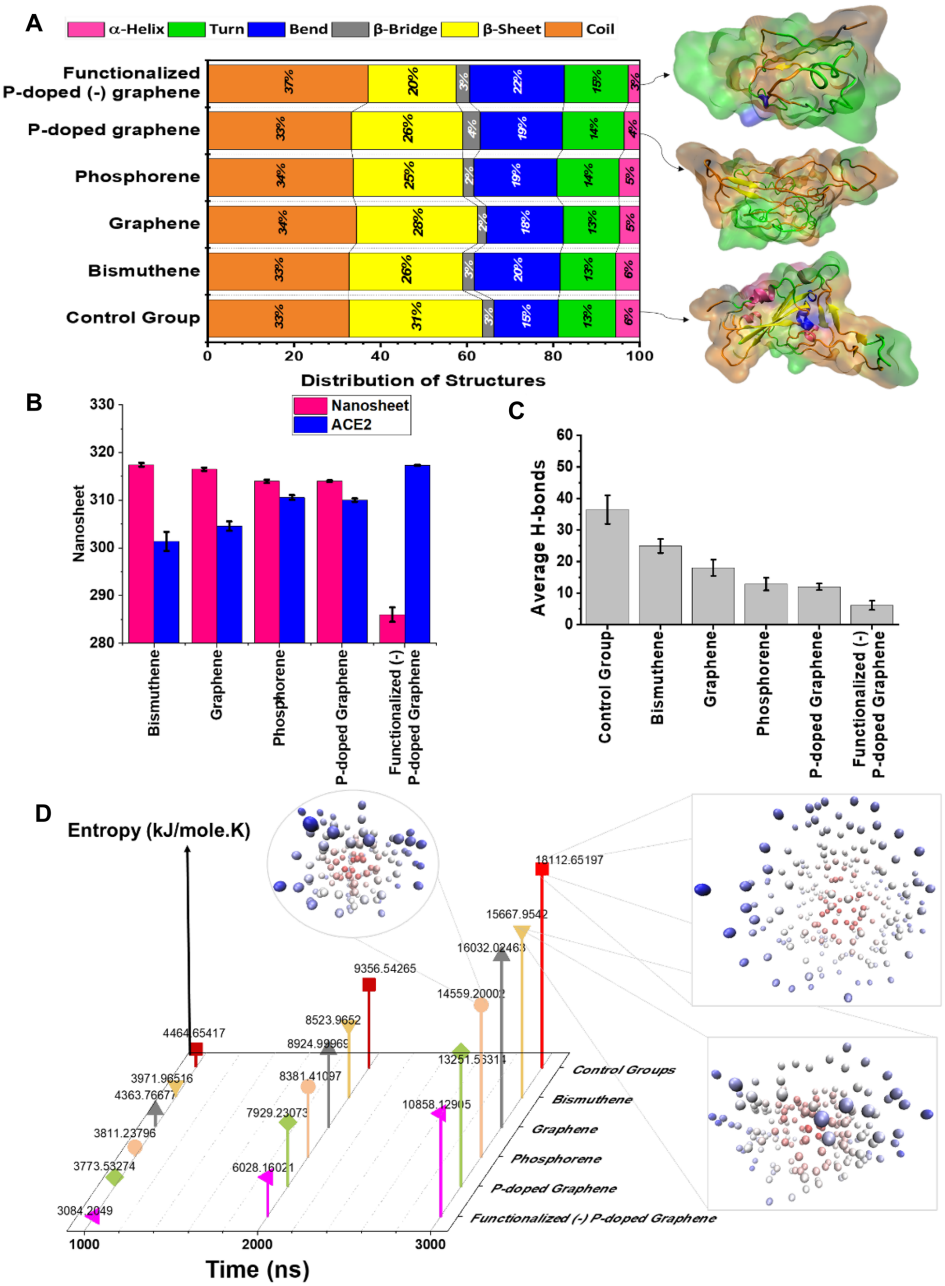
(**A**) Secondary structure distribution of spike protein demonstrates that the lowest amount of β-sheet belongs to the sample stimulated by phosphorene. (**B**) Solvent available surface area (SASA) of the protein with and without the impact of 2D nanomaterials. Clearly, the presence of phosphorene extends the protein toward the solvent. (**C**) Average H-bond counts between spike protein and ACE2 during the simulations. (**D**) Entropy analysis of ACE2-spike protein complexes in each case. Clearly, the maximum change in the entropy is the corresponding control group, while the lowest changes occurred for the ACE2-spike protein deformed by functionalized (-) p-doped graphene

Like other proteins, we can analyze the secondary structures of the spike protein of the SARS-CoV-2. The β-sheets and α-helices augment the stability of spike protein molecular structure, while coil, bend, and turn structures abate its stability. Figure 4A demonstrates the distribution of spike proteinʼs secondary structures after interaction with 2D nanomaterials. Snapshots of the structures are provided with the same color code as the diagram to provide better insight into the conformational analysis. The highest stability of the protein was observed in the case of the control group that is not deformed or distorted. Interestingly, the effect of p-doped graphene (26% of β-sheet and 5% of α-helix) was between graphene (28% of β-sheet and 5% of α-helix) and phosphorene (25% of β-sheet and 5% of α-helix). However, surface modification with carboxylate groups (20% of β-sheet and 3% of α-helix) augmented the instability within the structure. Therefore, it is understood that both the architecture of the nanomaterial and its surface chemistry determine its inhibitory effect against the virus.

The mean solvent accessible surface areas (SASA) of the spike protein-nanomaterial and spike protein-ACE2 pairs are presented in Fig. 4B. Increasing the interaction between 2D structures and spike protein resulted in the adsorption of this protein on the surface of 2D structures and reduction in their contact area with aqueous media. According to the results of SASA analysis, spike protein had the lowest contact area with aqueous media after interaction with functionalized (-) p-doped graphene, indicating the stronger interaction of spike protein with the nanomaterial, and thus, its greater effect on the deformation of the spike protein. Wang et al. [85] reported that hydrophobic interactions between spike protein and its receptor are determinants of the more transmissibility of SARS-CoV-2. Therefore, compounds that can abrupt the hydrophobic interactions between ACE2 and spike protein can be employed to combat COVID-19. In this regard, the effect of spike protein deformation by 2D structures on its interaction with ACE2 was investigated. After deformation and during the interaction with ACE2, spike protein is exposed toward aqueous media, which indicated the decreased hydrophobic interaction of the structures, both ACE2 and spike protein. As it is obvious, the largest SASA value is associated with the spike protein that is deformed by functionalized (-) p-doped graphene, indicating the exposure of spike protein to solvent molecules rather than the ACE2 receptor.

Hydrogen bonds (H-bonds) are also recognized as the main factor in the more contagion of SARS-CoV-2 as compared with previous coronavirus family members [85, 86]. Based on the results of a long time-scale simulation (400 ns), Ghorbani et al. [20] stated that SARS-CoV-2 forms H-bonds as double as the numbers that SARS-CoV (widespread in 2002) forms with ACE2. Therefore, it seems that prevention and reduction of H-bonds between ACE2 and SARS-CoV-2 can regulate its transmissibility and consequently harmful effect. Figure 4C shows the average H-bonds formed between the spike protein and ACE2 in the course of 3000 ns interactions. Although all 2D structures reduce the hydrogen bonds between spike protein and ACE2 by changing the structure, the functionalized (-)-p-doped-graphene-deformed spike protein reduces the average number of H-bonds with ACE2 from ca. 36.5 (pristine spike protein) to 6 (deformed protein) where using graphene or phosphorene the number drops only to 18 and 12, respectively. Therefore, surface engineering of 2D nanomaterials can significantly improve their antiviral performance. To confirm the effect of 2D nanomaterials on the deformation of spike protein and consequently deterioration of its interaction with ACE2, the entropy of deformed spike protein interaction with ACE2 was investigated using Schlitter’s entropy [84]. The higher difference between initial and final entropy displayed the stronger interaction of spike protein and ACE2. Figure 4D illustrates the mean entropy resulted from the interaction between the spike protein and ACE2 at different stages. The utilization of these 2D structures reduced the absolute energy and the difference between initial and final entropy. This results in the reduction of the absolute Gibbs free energy, showing the effectiveness of spike protein’s structure deformation by 2D nanostructures and consequently the hindrance of complex formation between spike protein and ACE2.

### Translocation of 2D nanomaterials through phospholipid membrane

As a result of the fact that the membrane supports the whole structure of the virus and especially keeps the spike protein in place to fuse into ACE2 [87], flexing and rupture of the membrane can interfere with the function of other parts, such as spike protein [88]. The membrane consists of hydrophilic, hydrophobic domains with carboxyl end groups that form ion channels [89]. It has been shown that lipid groups are the main components of the membranes [90, 91]. Hence, Eslami et al. [92] utilized the dipalmitoyl phosphatidylcholine (DPPC) lipid bilayer as the model for the SARS-CoV-2 membrane to investigate the effect of alcoholic disinfectants on the virus functions. Chen et al. [93] showed that modification of graphene surface to graphene oxide resulted in more attracted lipids toward the structure, which can be in favor of distorting the virus membrane. To view the interaction of nanomaterials with SARS-CoV-2, the penetration of them through a phospholipid membrane was simulated. In line with previous research [92], we also have utilized DPPC bilayer in the microsecond simulations to study the nanomaterialsʼ contacts with the virus through the phospholipid membrane. It is worth mentioning that due to the formation of ion channels through the membrane, together with carboxylate groups, amine groups (-NH_2_) are also being added onto the surface of p-doped graphene to have an array of positive functional groups alongside negative groups. Further surface modification is designed to improve the interaction of nanomaterials with DPPC.

**Fig. 5:**
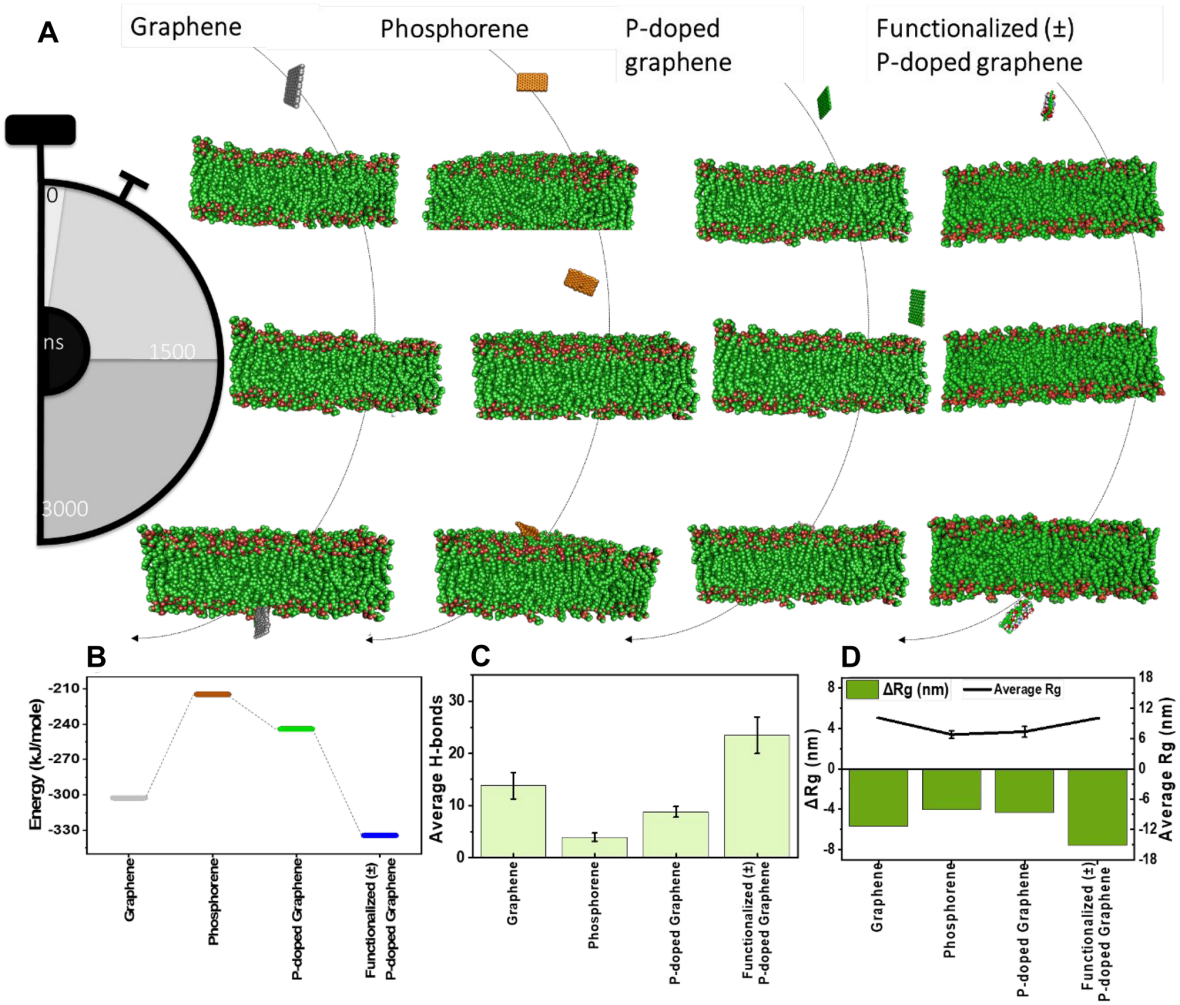
Impact of 2D nanomaterials on the phospholipid membrane of SARS-CoV-2 (Video S6). (**A**) Microsecond simulation of crossing nanomaterials through the phospholipid membrane. (**B**) Energy of the nanomaterial penetration through the membrane. (**C**) Average number of H-bonds formed between the membrane and nanomaterials. (**D**) Average and the difference in the Rg of the membrane that is observed due to the translocation of nano-objects in the phospholipid bilayer

Initial screening of results from the previous section showed that phosphorene, graphene, p-doped graphene, and functionalized p-doped graphene provide better interactions with spike protein. Therefore, these 2D nanomaterials are considered for further assessments in microsecond-long simulations (Fig. 5A). As it can be seen, graphene and functionalized p-doped graphene can easily penetrate through the membrane, while phosphorene is stuck on the membrane surface at the end of 3000 ns. Strikingly, p-doped graphene cannot also translocate through the membrane during the long-time interaction. Consistent with previous results [94], the insertion of graphene in the membrane is assisted through the interactions of the membranesʼ hydrophobic segments with carbon nanostructure. Ion channels and the presence of negative and positive functional groups are the main reasons for the easy translocation of functionalized p-doped graphene.

The average energy of the interaction over the course of simulations is quantitatively calculated and represented in Fig. 5B for the 2D nanomaterials. Consistent with the schematics of simulations, the most stable complex is corresponding to the functionalized (±) p-doped graphene. It can be attributed to the synergy of the hydrophobic and amplified electrostatic interactions, which cause easy and better penetration through the phospholipid membrane. Moreover, the H-bond formation between functional groups of nanomaterials and various domains of phospholipid membrane assists the interactions. In this line, it can be observed (Fig. 5C) that the highest number of H-bonds are formed in the case of functionalized (±) p-doped graphene due to the presence of –COOH and NH_2_ groups.

Figure 5D shows the changes in the radius of gyration, defined as the initial radius of gyration minus the final radius of gyration. The greater these changes, the greater the nanostructure attraction energy to adsorb the phospholipid membrane. As shown, the largest change in the radius of gyration was related to functionalized (±) p-doped graphene. As a result of its strong electrostatic attraction, this structure can well absorb phospholipid membranes and shrinks membrane molecules. Overall, it is clear that the proposed nanomaterials provide both successful interactions with spike protein and also their penetration through the phospholipid membrane bilayer seems promising, bringing the hope that they can be used to destabilize the whole SARS-CoV-2.

### 2D nanomaterials against SARS-CoV-2 M^pro^

To characterize more inhibitory potentials of proposed 2D nanomaterials against SARS-CoV-2, we set additional coarse-grained long time-scale simulations to elucidate their impact on SARS-CoV-2 M^pro^. Up to date, researchers have investigated various inhibitors against the M^pro^, such as peptide-inhibitors [57, 95, 96], previously FDA-approved drugs [97, 98], and herbal components [99]. Our observations from the previous section confirmed that 2D engineered nanomaterials are competent in blocking spike protein. These successful results encouraged us to consider their impact on the prevention of the infection propagation in a case that the virus can enter the body.

**Fig. 6:**
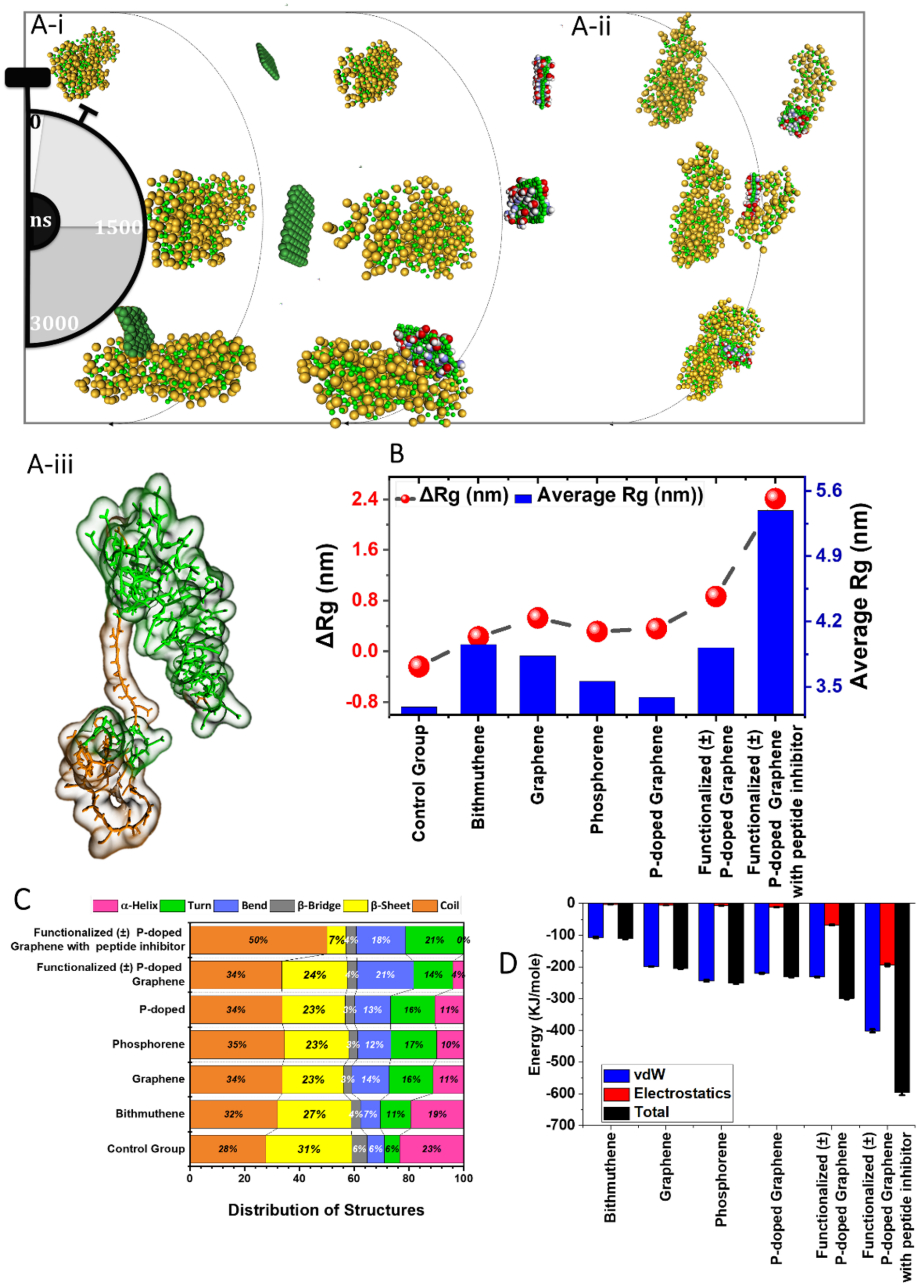
Effect of 2D materials on the SARS-CoV2 M^pro^ [22]. The interaction of p-doped graphene (left) and functionalized (±) p-doped graphene (right) with M^pro^ in 3-microsecond simulations. (**A-ii**) the interaction of functionalized (±) with peptide p-doped graphene during longer simulation time, 5000 ns. (**A-iii**) The structure of peptide inhibitor conjugated to nanomaterials surface. (**B**) Analysis of average Rg and the ΔRg for all cases. (**C**) Distribution of secondary structures for SARS-CoV-2 M^pro^ after distortion with 2D nanomaterials. (**D**) Energetic analysis of interactions with SARS-CoV-2 M^pro^. Surface decoration improves electrostatic attraction and consequently decreases the total energy level with p-doped graphene nanomaterial. Videos S7-S12 represent the corresponding analysis of the effect of 2D nanomaterials on the SARS-CoV2 M^pro^

Since the binding site of the M^pro^ structure includes hydrophobic, hydrophilic, and charged residues (positive and negative), in this stage, we decorated the p-doped surface alongside negative functional groups (carboxylate) with positive functional groups (amine) to amplify its performance against M^pro^. Figure 6A-i outlines various stages of simulations for only two cases (p-doped and functionalized (±) p-doped interactions with M^pro^). In a recent study, Han and Kral designed and investigated four different peptides composed of components from the virus-binding domains of ACE2 against SARS-CoV-2. They suggested that peptide group named inhibitor 3 can be used as a therapeutic for COVID-19. In this part, we designed another functionalized (±) p-doped graphene with a conjugated inhibitor3 on one side (due to the larger size of peptide compared with nanomaterial, only one inhibitor is designed on the surface). Figure 6A-ii represents three stages of simulation with inhibitor3-conjugated functionalized (±) p-doped graphene, and Fig. 6A-iii represents the inhibitor3 molecular structure. It should be mentioned that for the simulation of the interaction of inhibitor3-conjugated nanomaterial, time was set at 5000 ns to ensure sufficient time for the process.

The results of the initial evaluations are reported in Table 1. The results of the average root-mean-square-distribution (RMSD) and the average root-mean-square-fluctuation (RMSF) depict details on the stability of the M^pro^-nanomaterial complexes. The RMSD results show the extent of the M^pro^ in an extended configuration, which indicates its activity. Clearly, the nanomaterials capture the M^pro^ and reduce its activity. The lowest range of distribution is reported for the M^pro^/inhibitor3 conjugated-functionalized (±) p-doped graphene complex. Similarly, the RMSF results represent that the inhibitor3 conjugated-functionalized (±) p-doped graphene narrowed down the broad range of fluctuations (83.4 (Å) for the control group) to less than half (12.9 (Å)). Comparison between RMSD and RMSF results in the Table 1 and secondary structure distribution of M^pro^ exhibits a correlation between them, where more α-helical structures arise at higher RMSD and RMSF values. All of these findings confirm the excellent capability of the modified p-doped graphene in blocking M^pro^. It is worthy of mentioning that in all results reported in the table, the performance of p-doped graphene is between phosphorene and graphene. It can be attributed to the properties of p-doped graphene that inherit from its constituents (phosphor and carbon). The lowered SASA values illustrate the prevented exposure toward aqueous media and blockage of active sites that inhibit the infection spread throughout the body.

**Table 1:** The results of coarse-grained simulations for the interaction of nanomaterials with SARS-CoV-2 M^pro^

**Nanomaterial**	**Average RMSD (Å)**	**Average RMSF (Å)**	**Average SASA (nm**^**2**^**)**
**Control group**	51.6	8.34	354.32
**Bismuthene**	49.3	72.6	350.21
**Graphene**	48.1	58.1	345.74
**Phosphorene**	45.4	44.5	341.89
**P-doped graphene**	46.2	53.6	342.11
**Functionalized P-doped graphene**	43.6	39.5	338.61
**Functionalized (±) P-doped graphene with peptide inhibitor**	11.5	12.9	296.62

To elucidate the inhibitory mechanism of the nanomaterials, we considered more detail on the configuration and structure of M^pro^ after distortion phenomena. Figure 6B presents the average Rg and ΔRg observed in each case. Projections on XY and XZ planes are helpful to perceive the trend of change in the ΔRg and average Rg, respectively. The size of spheres exhibits a scale of the average Rg during the simulation; i.e., the smallest ball is related to the control group indicative of the packed structure of M^pro^ in comparison with its peers. Like spike protein, the reduced Rg size identifies the advanced stages of M^pro^ configurational changes during 3000 ns. Moreover, employing nanomaterials prevents M^pro^ from advancing to further stages. The observations validate the previous findings on the inhibitory effect of nanomaterials.

To shed more light on the configurational changes during the simulations, the secondary structure of M^pro^ in each simulation is reported in Fig. 6C. Apparently, the presence of nanomaterials increases the coil configuration and reduces the β-sheet and α-helix percentage in the secondary structure of the protein, which leads to destabilization of M^pro^. However, the α-helix percentage dropped from 11 to 4% with surface modifications where the presence of peptide conjugate drops it to zero. It demonstrates that the formation of α-helix in the structure of M^pro^ is blocked through the addition of peptide inhibitors. The β-sheet percentage drops from 31% in the control group to 7% in the presence of the inhibitor-conjugated functionalized (±) p-doped graphene. All the reported data confirm the excellent capability of a designed nanomaterial in the inactivation of the M^pro^.

A more plausible explanation for the observations can be provided through energetic evaluations. Comparison of energy levels (Fig. 6D) gives more insight into the surface decoration with functional groups. It can be seen that surface engineering of p-doped nanomaterial increases the electrostatic interaction energy from −11.47 kJ/mole to ca. −67.63 kJ/mole. This value even reached more than −200 kJ/mole with an inhibitor conjugated to the surface. It worth mentioning that with each surface engineering (addition of functional groups and peptide inhibitor), the electrostatic interaction had almost no significant contribution to the total energy. Moreover, surface modifications hinder the α-helix structure, meaning that the surface tuning has a critical role in the deactivation and destabilization of the virus. Moreover, H-bond formation was observed only between functionalized (±) nanomaterial and M^pro^, while the assessments showed no H-bond formation with other nanomaterials. During the simulations, ca. 20 H-bonds form between M^pro^ and functionalized (±) p-doped graphene, where the number hits 64 bonds during the simulation. These observations are attributed to the presence of –NH_2_ and –COOH groups and a long peptide chain that all offer potential positions for H-bond formation.

From the energetic point of view, the performance of p-doped graphene is between graphene and phosphorene, showing that it possesses both properties. Additional surface tuning improved inhibitory effect of 2D nanomaterials through boasted electrostatic interactions and H-bond formation. Therefore, considering the total energy of the complex, functionalized p-doped graphene was considered the most suitable nanomaterial to attack the M^pro^ and destabilize the secondary structure of the protein.

## Conclusions

The attachment of SARS-CoV-2ʼs spike protein to ACE2 is the key step for the activity of the virus in the body. Restructuring the spike protein and preventing it from binding to ACE2 is one of the best approaches to disrupt the pathogenesis of SARS-CoV-2. In the first stage, the deforming effects of 2D structures on spike protein were investigated. Outcomes from this step showed that the architecture and surface engineering of the nanomaterials plays a critical role in the inhibitory effect of 2D nanomaterials against SARS-CoV-2. Afterward, pristine spike protein and distorted spike protein were brought together to interact with ACE2 in order to quantitatively monitor and compare the inhibitory effects of tunable structures. The evaluations were performed through powerful tools of molecular dynamics and docking simulation. Although the spike protein was deformed by all types of 2D nanomaterials and it showed less affinity to interact with ACE2, the deformation of the spike protein by functionalized (-) p-doped graphene resulted in the following: (i) the lowest absolute energy, (ii) the lowest compactness, (iii) the highest contact area with aqueous media, and (iv) the most fluctuations, making it the best 2D nanomaterials among others to stop the replication of SARS-CoV-2 at the infection site. Unal et al. evaluated the effects of graphene oxide nanosheets on the S protein of SARS-CoV-2 both in silico and in vitro*.* The results from in vitro study showed that graphene oxide nanosheets disrupted infectivity, which was consistent with their molecular docking results. Also they suggested further surface functionalization of graphene oxide nanosheets, which is in agreement with our results. In due course, another set of long time-scale simulations were performed to monitor the impact of the proposed nanomaterials on the M^pro^ of SARS-CoV-2. The results confirmed the competency of 2D nanomaterials in deactivating M^pro^ and avoidance of infection spread. Overall, this work demonstrates that 2D nanomaterials can be considered preventive agents against the transmissibility and the activity of SARS-CoV-2.

## Supplementary Information

Below is the link to the electronic supplementary material.

Supplementary file1 (RAR 60586 KB)

Supplementary file2 (DOCX 917 KB)

## Data Availability

The authors confirm that the data supporting the findings of this study are available within the article and its supplementary materials. Data will be available on the request from the authors.

## References

[1] Nikaeen G, Abbaszadeh S, Yousefinejad S (2020). Application of nanomaterials in treatment, anti-infection and detection of coronaviruses. Nanomedicine.

[2] Tremiliosi GC, Simoes LGP, Minozzi DT, Santos RI, Vilela DCB, Durigon EL, et al. Ag nanoparticles-based antimicrobial polycotton fabrics to prevent the transmission and spread of SARS-CoV-2. BioRxiv. 2020. 06.26.152520. 10.1101/2020.06.26.152520

[3] Chen Z, Zhang Z, Zhai X, Li Y, Lin L, Zhao H (2020). Rapid and sensitive detection of anti-SARS-CoV-2 IgG, using lanthanide-doped nanoparticles-based lateral flow immunoassay. Anal Chem.

[4] Moitra P, Alafeef M, Dighe K, Frieman MB, Pan D (2020). Selective naked-eye detection of SARS-CoV-2 mediated by N gene targeted antisense oligonucleotide capped plasmonic nanoparticles. ACS Nano.

[5] Jeremiah SS, Miyakawa K, Morita T, Yamaoka Y, Ryoa A (2020). Potent antiviral effect of silver nanoparticles on SARS-CoV-2. Biochem Biophys Res Commun.

[6] Yao H, Song Y, Chen Y, Wu N, Xu J, Sun C (2020). Molecular architecture of the SARS-CoV-2 virus. Cell.

[7] McKay PF, Hu K, Blakney AK, Samnuan K, Brown JC, Penn R (2020). Self-amplifying RNA SARS-CoV-2 lipid nanoparticle vaccine candidate induces high neutralizing antibody titers in mice. Nat Commun.

[8] Raghuwanshi D, Mishra V, Das D, Kaur K, Suresh MR (2012). Dendritic cell targeted chitosan nanoparticles for nasal DNA immunization against SARS CoV nucleocapsid protein. Mol Pharm.

[9] Raghuwanshi D, Mishra V, Das D, Kaur K, Suresh MR (2020). 2D nanomaterials for cancer theranostic applications. Adv Mater.

[10] Guo Z, Ouyang J, Kim NY, Shi J, Ji X (2019). Emerging two-dimensional nanomaterials for cancer therapy. ChemPhysChem.

[11] Liu S, Pan X, Liu H (2020). Two-dimensional nanomaterials for photothermal therapy. Angew Chem.

[12] Hu T, Mei X, Wang Y, Weng X, Liang R, Wei M (2019). Two-dimensional nanomaterials: fascinating materials in biomedical field. Sci.

[13] Wen W, Song Y, Yan X, Zhu C, Du D, Wang S (2018). Recent advances in emerging 2D nanomaterials for biosensing and bioimaging applications. Mater Today.

[14] Wang L, Xiong Q, Xiao F, Duan H (2017). 2D nanomaterials based electrochemical biosensors for cancer diagnosis. Biosens Bioelectron.

[15] Zhang J, Chen H, Zhao M, Liu G, Wu J (2020). 2D nanomaterials for tissue engineering application. Nano Res.

[16] Arantes PR, Saha A, Palermo G (2020). Fighting COVID-19 using molecular dynamics simulations. ACS Cent Sci.

[17] Walls AC, Park YJ, Tortorici MA, Wall A, McGuire AT, Veesler D (2020). Structure function and antigenicity of the SARS-CoV-2 spike glycoprotein. Cell.

[18] Bar-On YM, Flamholz A, Phillips R, Milo R. Science Forum: SARS-CoV-2 (COVID-19) by the numbers. Elife. 2020;9:e57309. 10.7554/eLife.57309 PMC7224694 32228860

[19] Lan J, Ge J, Yu J, Shan S, Zhou H, Fan S (2020). Structure of the SARS-CoV-2 spike receptor-binding domain bound to the ACE2 receptor. Nature.

[20] Ghorbani M, Brooks BR, Klauda JB (2020). Critical sequence hotspots for binding of novel coronavirus to angiotensin converter enzyme as evaluated by molecular simulations. J Phys Chem B.

[21] Ke Z, Oton J, Qu K, Cortese M, Zila V, McKeane L (2020). Structures and distributions of SARS-CoV-2 spike proteins on intact virions. Nature.

[22] García-Iriepa C, Hognon C, Francés-Monerris A, Iriepa I, Miclot T, Barone G, et al. Thermodynamics of the interaction between the spike protein of severe acute respiratory syndrome coronavirus-2 and the receptor of human angiotensin-converting enzyme 2. Effects of Possible Ligands. J Phys Chem Lett. 2020;11:9272–81. 10.1021/acs.jpclett.0c02203 33085491

[23] Basu A, Sarkar A, Maulik U (2020). Molecular docking study of potential phytochemicals and their effects on the complex of SARS-CoV2 spike protein and human ACE2. Sci Rep.

[24] Yu JW, Wang L, Bao LD. Exploring the active compounds of traditional Mongolian medicine in intervention of novel coronavirus (COVID-19) based on molecular docking method. J Funct Foods. 2020;71:104016. 10.1016/j.jff.2020.104016 PMC7225714 32421102

[25] Pandey P, Rane JS, Chatterjee A, Kumar A, Khan R, Prakash A, et al. Targeting SARS-CoV-2 spike protein of COVID-19 with naturally occurring phytochemicals: an in silico study for drug development. J Biomol Struct Dyn. 2020;1–11. 10.1080/07391102.2020.1796811 PMC7441770 32698689

[26] Ling R, Dai Y, Huang B, Huang W, Yu J, Lu X, et al. In silico design of antiviral peptides targeting the spike protein of SARS-CoV-2. Peptides. 2020;130:170328. 10.1016/j.peptides.2020.170328 PMC7198429 32380200

[27] Han Y, Král P (2020). Computational design of ACE2-based peptide inhibitors of SARS-CoV-2. ACS Nano.

[28] Gorshkov K, Susumu K, Chen J, Xu M, Pradhan M, Zhu W (2020). Quantum dot-conjugated SARS-CoV-2 spike pseudo-virions enable tracking of angiotensin converting enzyme 2 binding and endocytosis. ACS Nano.

[29] Gupta MK, Vemula S, Donde R, Gouda G, Behera L, Vadde R (2021). In-silico approaches to detect inhibitors of the human severe acute respiratory syndrome coronavirus envelope protein ion channel. J Biomol Struct Dyn.

[30] Fiani B, Covarrubias C, Desai A, Sekhon M, Jarrah R (2020). A contemporary review of neurological sequelae of COVID-19. Front Neurol..

[31] Kordzadeh A, Saadatabadi AR, Hadi A (2020). Investigation on penetration of saffron components through lipid bilayer bound to spike protein of SARS-CoV-2 using steered molecular dynamics simulation. Heliyon.

[32] Zucker I, Werber JR, Fishman ZS, Hashmi SM, Gabinet UR, Lu X (2017). Loss of phospholipid membrane integrity induced by two-dimensional nanomaterials. Environ Sci Technol Lett.

[33] Kampf G, Todt D, Pfaender S, Steinmann E (2020). Persistence of coronaviruses on inanimate surfaces and their inactivation with biocidal agents. J Hosp Infect.

[34] Workman AD, Welling DB, Carter BS, Curry WT, Holbrook EH, Gray ST (2020). Endonasal instrumentation and aerosolization risk in the era of COVID-19: simulation literature review, and proposed mitigation strategies. Int Forum Allergy Rhinol.

[35] Leung NHL, Chu DKW, Shiu EYC, Chan KH, McDevitt JJ, Hau BJP (2020). Respiratory virus shedding in exhaled breath and efficacy of face masks. Nat Med.

[36] Cook T (2020). Personal protective equipment during the coronavirus disease (COVID) 2019 pandemic–a narrative review. Anaesthesia.

[37] Leung CC, Lam TH, Cheng KK (2020). Mass masking in the COVID-19 epidemic: people need guidance. Lancet.

[38] Konda A, Prakash A, Moss GA, Schmoldt M, Grant GD, Guha S (2020). Aerosol filtration efficiency of common fabrics used in respiratory cloth masks. ACS Nano.

[39] Lin Z, Wang Z, Zhang X, Diao D (2021). Superhydrophobic, photo-sterilize, and reusable mask based on graphene nanosheet-embedded carbon (GNEC) film. Nano Res.

[40] El-Atab N, Qaiser N, Badghaish H, Shaikh SF, Hussain MM (2020). Flexible nanoporous template for the design and development of reusable anti-COVID-19 hydrophobic face masks. ACS Nano.

[41] Zhong H, Zhu Z, Lin J, Cheung CF, Lu VL, Yan F (2020). Reusable and recyclable graphene masks with outstanding superhydrophobic and photothermal performances. ACS Nano.

[42] Shan X, Zhang H, Liu C, Yu L, Di Y, Zhang X (2020). Reusable self-sterilization masks based on electrothermal graphene filters. ACS Appl Mater Interfaces.

[43] Srivastava AK, Dwivedi N, Dhand C, Khan R, Sathish N, Gupta MK, et al. Potential of graphene-based materials to combat COVID-19: properties perspectives and prospects. Mater Today Chem. 2020;18:100385. 10.1016/j.mtchem.2020.100385 PMC7577689 33106780

[44] Palmieri V, Papi M. Can graphene take part in the fight against COVID-19?. Nano Today. 2020;33:100883. 10.1016/j.nantod.2020.100883 PMC7203038 32382315

[45] Jiang Z, Feng B, Xu J, Qing T, Zhang P, Qingb Z. Graphene biosensors for bacterial and viral pathogens. Biosens Bioelectron. 2020;166:112471. 10.1016/j.bios.2020.112471 PMC7382337 32777726

[46] Ba H, Truong-Phuoc L, Papaefthimiou V, Sutter C, Pronkin S, Bahouka A (2020). Cotton fabrics coated with few-layer graphene as highly responsive surface heaters and integrated lightweight electronic-textile circuits. ACS Appl Nano Mater.

[47] Maio FD, Palmieri V, Babini G, Augello A, Palucci I, Perini G, et al. Graphene nanoplatelet and Graphene oxide functionalization of face mask materials inhibits infectivity of trapped SARS-CoV-2. Iscience. 2021;24(7):102788. 10.1016/j.isci.2021.102788 PMC8233064 34222841

[48] Seo G, Lee G, Kim MJ, Baek S, Choi M, Ku KB (2020). Rapid detection of COVID-19 causative virus (SARS-CoV-2) in human nasopharyngeal swab specimens using field-effect transistor-based biosensor. ACS Nano.

[49] Ménard-Moyon C, Bianco A, Kalantar-Zadeh K (2020). Two-dimensional material-based biosensors for virus detection. ACS Sens.

[50] Song S, Shen H , Wang Y, Chu X, Xie J, Zhou N, et al. Biomedical application of graphene: from drug delivery tumor therapy to theranostics. Colloids Surf B Biointerfaces. 2020; 185:110596. 10.1016/j.colsurfb.2019.110596 31707226

[51] Yang Y, Asiri AM, Tang Z, Du D, Lin Y (2013). Graphene based materials for biomedical applications. Mater Today.

[52] Tatullo M, Genovese F, Aiello E, Amantea M, Makeeva I, Zavan B (2019). Phosphorene is the new graphene in biomedical applications. Materials.

[53] Zhang W, Huynh T, Xiu P, Zhou B, Ye C, Luan B (2015). Revealing the importance of surface morphology of nanomaterials to biological responses: Adsorption of the villin headpiece onto graphene and phosphorene. Carbon.

[54] Han M, Zhu L, Mo J, Wei W, Yuan B, Zhao J (2020). Protein corona and immune responses of borophene: a comparison of nanosheet–plasma interface with graphene and phosphorene. ACS Appl Bio Mater.

[55] Bhuvaneswari R, Nagarajan V, Chandiramouli R. Electronic properties of novel bismuthene nanosheets with adsorption studies of G-series nerve agent molecules – a DFT outlook. Phys Lett A. 2019;383:125975.

[56] Shi J, Sivaraman J, Song J (2008). Mechanism for controlling the dimer-monomer switch and coupling dimerization to catalysis of the severe acute respiratory syndrome coronavirus 3C-like protease. J Virol.

[57] Pant S, Singh M, Ravichandiran V, Murty USN, Srivastava HK (2021). Peptide-like and small-molecule inhibitors against Covid-19. J Biomol Struct Dyn.

[58] Verma N, Henderson JA, J Shen. Proton-coupled conformational activation of SARS coronavirus main proteases and opportunity for designing small-molecule broad-spectrum targeted covalent inhibitors. J Am Chem Soc. 2020;142:21883–90 10.1021/jacs.0c10770 PMC7754784 33320670

[59] Wan H, Aravamuthan V, Pearlstein RA (2020). Probing the dynamic structure–function and structure-free energy relationships of the coronavirus main protease with biodynamics theory. ACS Pharmacol Transl Sci.

[60] Wang J (2020). Fast identification of possible drug treatment of coronavirus disease-19 (COVID-19) through computational drug repurposing study. J Chem Inf Model.

[61] Xu Z, Yang L, Zhang X, et al. Discovery of potential flavonoid inhibitors against COVID-19 3CL proteinase based on virtual screening strategy. Front Mol Biosci. 2020;7:556481 10.3389/fmolb.2020.556481 PMC7561382 33134310

[62] Farag A, Wang P, Ahmed M, Sadek H (2020). Identification of FDA approved drugs targeting COVID-19 virus by structure-based drug repositioning.

[63] Vishnoi P, Pramoda K, Rao C (2019). 2D Elemental nanomaterials beyond graphene. Chem Nano Mat.

[64] Sha Z, Pei Q, Ding Z, Jiang J, Zhang Y. Mechanical properties and fracture behavior of single-layer phosphorene at finite temperatures. J Phys D. 2015;48:395303.

[65] Akhtar M, Anderson G, Zhao R, Alruqi A, Mroczkowska JE, Sumanasekera G, et al. Recent advances in synthesis properties and applications of phosphorene. NPJ 2D Mater Appl. 2017;1:1–13.

[66] Chabi S, Kadel K (2020). Two-dimensional silicon carbide: emerging direct band gap semiconductor. Nanomaterials.

[67] Hanwell MD, Curtis DE, Lonie DC, Vandermeersch T, Zurek E, Hutchison GR (2012). Avogadro: an advanced semantic chemical editor visualization and analysis platform. J Cheminformatics.

[68] Kühne TD, Iannuzzi M, Ben MD, Rybkin VV, Seewald P, Stein F, et al. CP2K: an electronic structure and molecular dynamics software package - Quickstep: Efficient and accurate electronic structure calculations. J Chem Phys. 2020;152:194103. 10.1063/5.0007045 33687235

[69] Jo S, Kim T, Iyer VG, Im W (2008). CHARMM-GUI: A web-based graphical user interface for CHARMM. J Comput Chem.

[70] Spoel DVD, Lindahl E, Hess B, Groenhof G, Mark AE, Berendsen HJC (2005). GROMACS: fast flexible and free. J Comput Chem.

[71] Sohraby F, Soltanabad MH, Bagheri M, Javan MB, Moghadam MJ, Baghkheirati EK (2020). Application of molecular dynamics in coating Ag-conjugated nanoparticles with potential therapeutic applications. Nano Biomed Eng.

[72] Li B, Hong S, Zhang X, Xiong C, Zhao G, Yang Q (2019). Understanding interfacial mechanics and mechanisms of exfoliation and stabilization of graphene using urea/glycerol solvents. Adv Theory Simul.

[73] Zandi P, Ghasemy E, Khedri M, Rashidi A, Maleki R, Jahromi AM (2021). Shedding light on miniaturized dialysis using MXene 2D materials: a computational chemistry approach. ACS Omega.

[74] Alimohammadi E, Khedri M, Miri Jahromi A, Maleki R, Rezaian M (2020). Graphene-based nanoparticles as potential treatment options for Parkinson's disease: a molecular dynamics study. Int J Nanomedicine.

[75] Marrink SJ, Risselada HJ, Yefimov S, Tieleman DP, de Vries AH (2007). The MARTINI force field: coarse grained model for biomolecular simulations. J Phys Chem B.

[76] Man VH, Li MS, Wang J, Derreumaux P, Nguyen PH. Interaction mechanism between the focused ultrasound and lipid membrane at the molecular level. J Chem Phys. 2019;150:215101. 10.1063/1.5099008 PMC7043851 31176320

[77] Hoffmann C, Centi A, Menichetti R, Bereau T (2020). Molecular dynamics trajectories for 630 coarse-grained drug-membrane permeations. Sci Data.

[78] Rajagopal N, Nangia S (2019). Obtaining protein association energy landscape for integral membrane proteins. J Chem Theory Comput.

[79] Goliaei A, Adhikari U, Berkowitz ML (2015). Opening of the blood-brain barrier tight junction due to shock wave induced bubble collapse: a molecular dynamics simulation study. ACS Chem Neurosci.

[80] Domicevica L, Koldsø H, Biggin PC (2018). Multiscale molecular dynamics simulations of lipid interactions with P-glycoprotein in a complex membrane. J Mol Graph Model.

[81] Maleki R, Khedri M, Rezvantalab S, Afsharchi F, Musaie K, Shafiee S (2021). β-amyloid targeting with two-dimensional covalent organic frameworks: multi-scale in-silico dissection of nano-biointerface. Chem Bio Chem.

[82] Trott O, Olson AJ (2010). AutoDock Vina: improving the speed and accuracy of docking with a new scoring function efficient optimization and multithreading. J Comput Chem.

[83] Takahashi T, Mihara H (2008). Peptide and protein mimetics inhibiting amyloid β-peptide aggregation. Acc Chem Res.

[84] Lee TH, Kim SY, Jang HW (2016). Black phosphorus: critical review and potential for water splitting photocatalyst. Nanomaterials.

[85] Wang Y, M Liu, J Gao. Enhanced receptor binding of SARS-CoV-2 through networks of hydrogen-bonding and hydrophobic interactions. Proc Natl Acad Sci. 2020;117:13967. 10.1073/pnas.2008209117 PMC7322019 32503918

[86] Spinello A, Saltalamacchia A, Magistrato A (2020). Is the rigidity of SARS-CoV-2 spike receptor-binding motif the hallmark for its enhanced infectivity? Insights from all-atom simulations. J Phys Chem Lett.

[87] Fiani B, Covarrubias C, Desai A, Sekhon M, Jarrah R (2020). A contemporary review of neurological sequelae of COVID-19. Front Neurol.

[88] Kordzadeh A, Saadatabadi AR, Hadi A. Investigation on penetration of saffron components through lipid bilayer bound to spike protein of SARS-CoV-2 using steered molecular dynamics simulation. Heliyon. 2020;6:e05681. 10.1016/j.heliyon.2020.e05681 PMC7733551 33344790

[89] Schoeman D, Fielding BC (2019). Coronavirus envelope protein: current knowledge. Virol J.

[90] Verdiá-Báguena C, Nieto-Torres JL, Alcaraz A, DeDiego ML, L Enjuanes, Aguilella VM. Analysis of SARS-CoV E protein ion channel activity by tuning the protein and lipid charge. Biochim Biophys Acta Biomembr. 2013;1828:2026–31. 10.1016/j.bbamem.2013.05.008 PMC3715572 23688394

[91] Verdiá-Báguena C, Nieto-Torres JL, Alcaraz A, DeDiego ML, Torres J, Aguilella VM (2012). Coronavirus E protein forms ion channels with functionally and structurally-involved membrane lipids. Virology.

[92] Eslami H, Das S, Zhou T, Müller-Plathe F (2020). How alcoholic disinfectants affect coronavirus model membranes: membrane fluidity permeability and disintegration. J Phys Chem B.

[93] Chen J, Zhou G, Chen L, Wang Y, Wang X, Zeng S (2016). Interaction of graphene and its oxide with lipid membrane: a molecular dynamics simulation study. J Phys Chem C.

[94] Puigpelat E, Ignés-Mullol J, Sagués F, Reigada R (2019). Interaction of graphene nanoparticles and lipid membranes displaying different liquid orderings: a molecular dynamics study. Langmuir.

[95] Zhang L, Lin D, Sun X, Curth U, Drosten C, Sauerhering L (2020). Crystal structure of SARS-CoV-2 main protease provides a basis for design of improved α-ketoamide inhibitors. Science.

[96] Amin SA, Banerjee S, Ghosh K, Gayen S, Jha T. Protease targeted COVID-19 drug discovery and its challenges: Insight into viral main protease (Mpro) and papain-like protease (PLpro) inhibitors. Bioorg Med Chem. 2021;29:115860. 10.1016/j.bmc.2020.115860 PMC7647411 33191083

[97] Shitrit A, Zaidman D, Kalid O, Bloch I, Doron D, Yarnizky T (2020). Conserved interactions required for in vitro inhibition of the main protease of severe acute respiratory syndrome coronavirus 2 (SARS-CoV-2). Sci Rep.

[98] Elmezayen AD, Al-Obaidi A, Şahin AT, Yelekçi K (2021). Drug repurposing for coronavirus (COVID-19): in silico screening of known drugs against coronavirus 3CL hydrolase and protease enzymes. J Biomol Struct Dyn.

[99] Ghosh R, Chakraborty A, Biswas A, Chowdhuri S. Evaluation of green tea polyphenols as novel corona virus (SARS CoV-2) main protease (Mpro) inhibitors - an in silico docking and molecular dynamics simulation study. J Biomol Struct Dyn. 2020;1–13. 10.1080/07391102.2020.1779818 PMC7332865 32568613

