# Supplementary material for: Engineering of 2D nanomaterials to trap and kill SARS-CoV-2: a new insight from multi-microsecond atomistic simulations
Source: Drug Deliv Transl Res. 2021 Sep 3;12(6):1408–22. doi: 10.1007/s13346-021-01054-w (PMC8413075; doi:10.1007/s13346-021-01054-w)
Supplement: Supplementary file 2 — Supplementary file2 (DOCX 917 KB) [file 13346_2021_1054_MOESM2_ESM.docx]

**Supporting Information**

**Engineering of 2D materials to trap and kill SARS-CoV-2: a new insight from multi-microsecond atomistic simulations**

Mohammad Khedri,^a,b,†^ Reza Maleki,^b,†^ Mohammad Dahri,^b,c^ Mohammad Moein Sadeghi,^b,c^ Sima Rezvantalab^d,*^, Hélder A. Santos,^a,e,^* Mohammad-Ali Shahbazi,^a,f,^*

^a^ Drug Research Program, Division of Pharmaceutical Chemistry and Technology, Faculty of Pharmacy, University of Helsinki, FI-00014 Helsinki, Finland

^b^ Computational Biology and Chemistry Group (CBCG), Universal Scientific Education and Research Network (USERN), Tehran, Iran

^c^ Student Research Committee, Shiraz University of Medical Sciences, Shiraz, Iran

^d^ Renewable Energies Department, Faculty of Chemical Engineering, Urmia University of Technology, 57166-419 Urmia, Iran

^e^ Helsinki Institute of Life Science (HiLIFE), University of Helsinki, FI-00014 Helsinki, Finland

^f^ Zanjan Pharmaceutical Nanotechnology Research Center (ZPNRC), Zanjan University of Medical Sciences, 45139-56184 Zanjan, Iran

* Corresponding Authors:

[s.rezvantalab@uut.ac.ir](mailto:s.rezvantalab@uut.ac.ir) (S. Rezvantalab),

[helder.santos@helsinki.fi](mailto:helder.santos@helsinki.fi) (H.A. Santos),

[m.a.shahbazi@helsinki.fi](mailto:m.a.shahbazi@helsinki.fi) (M.-A. Shahbazi)

† These authors have contributed equally to this work

**Contents**

**Figure S1.** 2D nanomaterials used in the interaction with spike protein and M^pro^.

**Figure S2.** Analysis of interaction of residues in the spike protein with nanomaterials.

**Figure S3.** Mapping Rg vs. energy of binding for bismuthene and graphene.

**Figure S4.** (A-B) Comparison between simulation of Han et al. [1] and repeated simulations. (C) Comparison between experimental results of Pramanik et al. [2] and simulations results that are performed in this work. (D) Comparison between experimental results of Huang et al. [3] and simulations results performed in this work.

**Table S1.** Error bars of Energy for Nanostructures and spike protein.


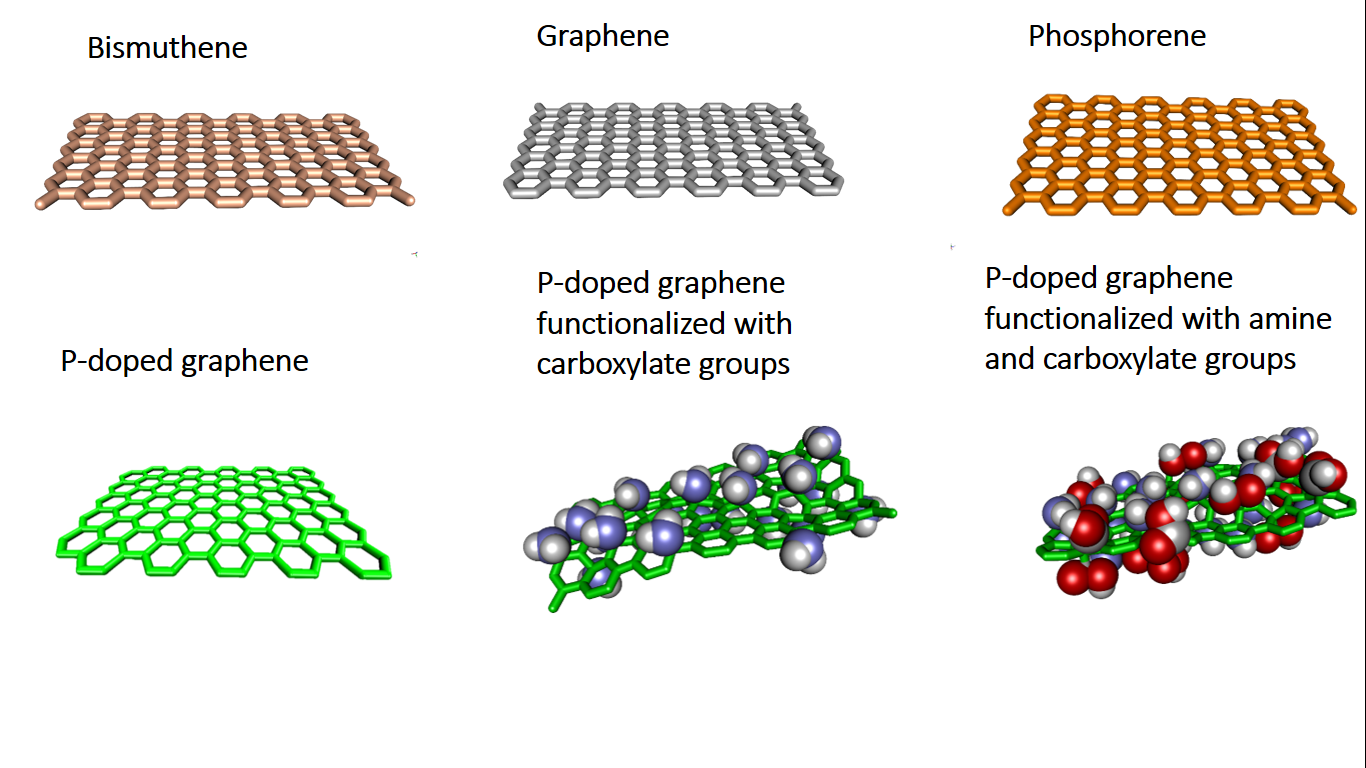


**Figure S1.** 2D structures used in the interaction with spike protein and M^pro^.

**Validation**

To confirm the simulation results, the simulations performed by Han *et al*. [1] was replicated using GROMACS software and CHARMM36 force field. **Figure S2.A- B** compares the results of repeated simulations with the results that are shown in **Figure 2.f** of Han *et al*. The similarity of the RMSD values obtained indicates the accuracy of the simulations performed in this work.

The experiments performed by Pramanik *et al*. [2] were also simulated using GROMACS software. To do this, the interactions between angiotensin-converting enzyme 2 (ACE2) and spike protein in the presence of gold nanoparticles (GNPs), anti-spike antibody and buffer (Mock) was investigated. In these simulations, the OPLSA force field is used. The lower the absolute value of the energy resulting from the interactions of ACE2 and spike protein, the greater the effect of nanoparticles in preventing virus infection. In this regard, **Figure S2.C** shows the energy obtained from the interaction of ACE2 and spike protein and the results of Pramanik *et al*.ʼs work (**Figure 4.c** of the article). According to the results, the least interactions of ACE2 and spike protein occurred in the presence of GNP + 100 ng antibody. The consistency of the simulations and the results of the laboratory work of Pramanik *et al*. shows the correctness of the algorithms and simulation methods used in this work.

Also, Huang *et al*. [3] investigated the effect of pregnancy-induced hypertension (PIH) and gold nanorod complex (PIH-AuNRs) in inhibition of Middle East respiratory syndrome coronavirus (MERS-CoV) fusion in the Huh-7 cell. In this regard, interactions of MERS-CoV and Huh-7 in the presence of PIH and PIH-AuNRs is simulated using GROMACS software and OPLSA forcefield. The reduction of the absolute value of the MERS-CoV and Huh-7 interaction indicates the greater effect of the nanoparticles in inhibition of MERS-CoV fusion in the Huh-7 cell. **Figure S2.D** shows the energy of MERS-CoV and Huh-7 interactions and the results from the laboratory work of Huang *et al*. (**Figure 5.g** of Huang *et al*.’s article). The simulation results, like the results obtained from the work of Huang *et al*. [3], Show a decrease in MERS-CoV and Huh-7 interactions in the presence of PIH-AuNRs. This also shows the accuracy of algorithms and simulation methods.


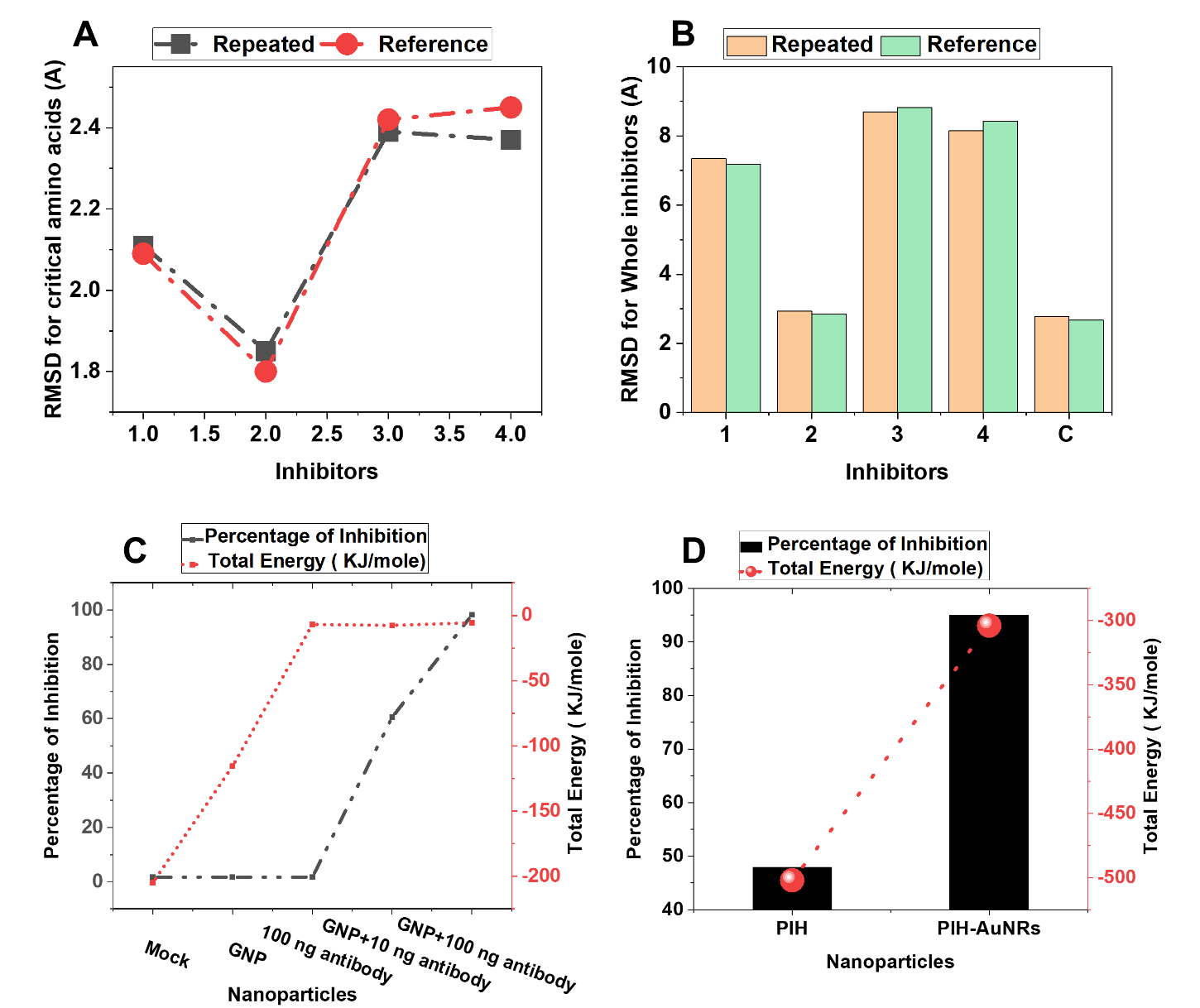


**Figure S2.** (A-B) Comparison between simulation of Han *et al*. [1] and repeated simulations. (C) Comparison between experimental results of Pramanik *et al*. [2] and simulations results that are performed in this work. (D) Comparison between experimental results of Huang *et al*. [3] and simulationsʼ results performed in this work.


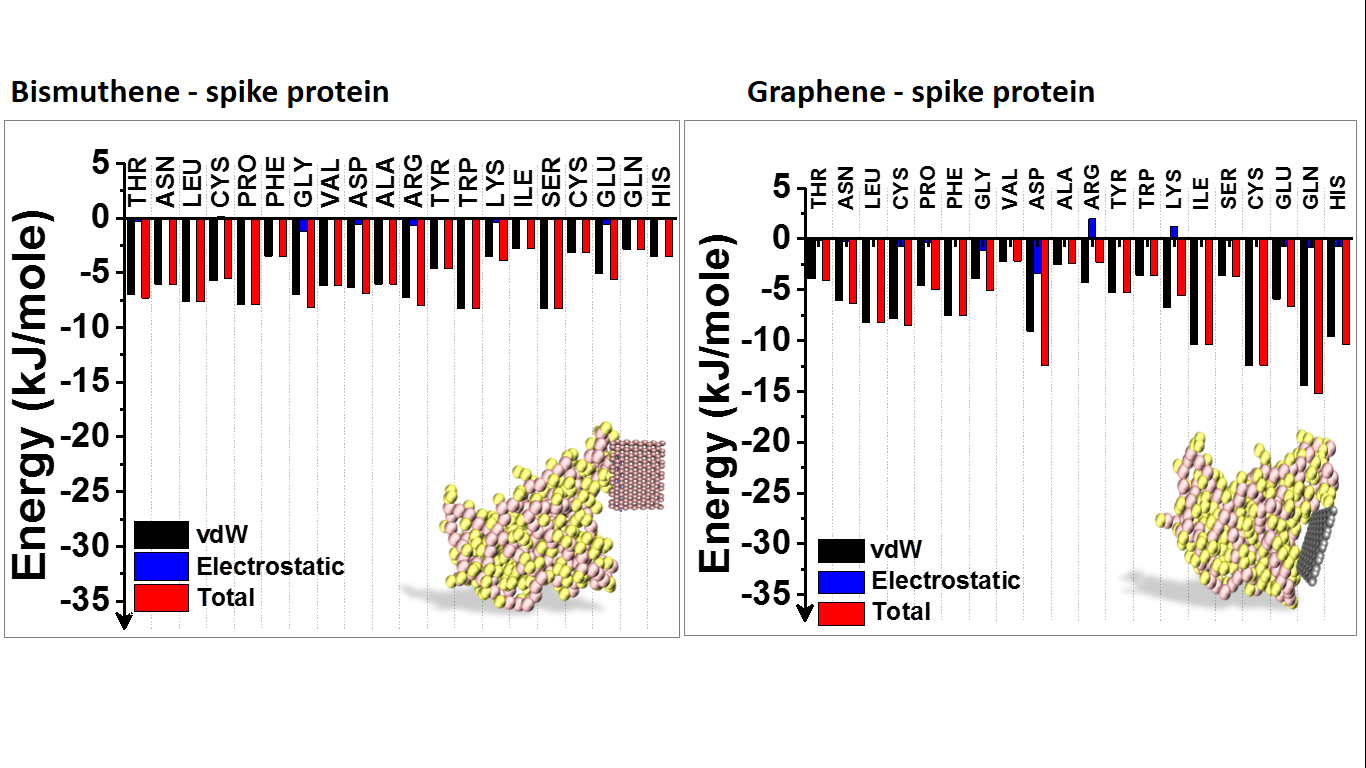


**Figure S3.** Analysis of interaction of residues in the spike protein with nanosheets.


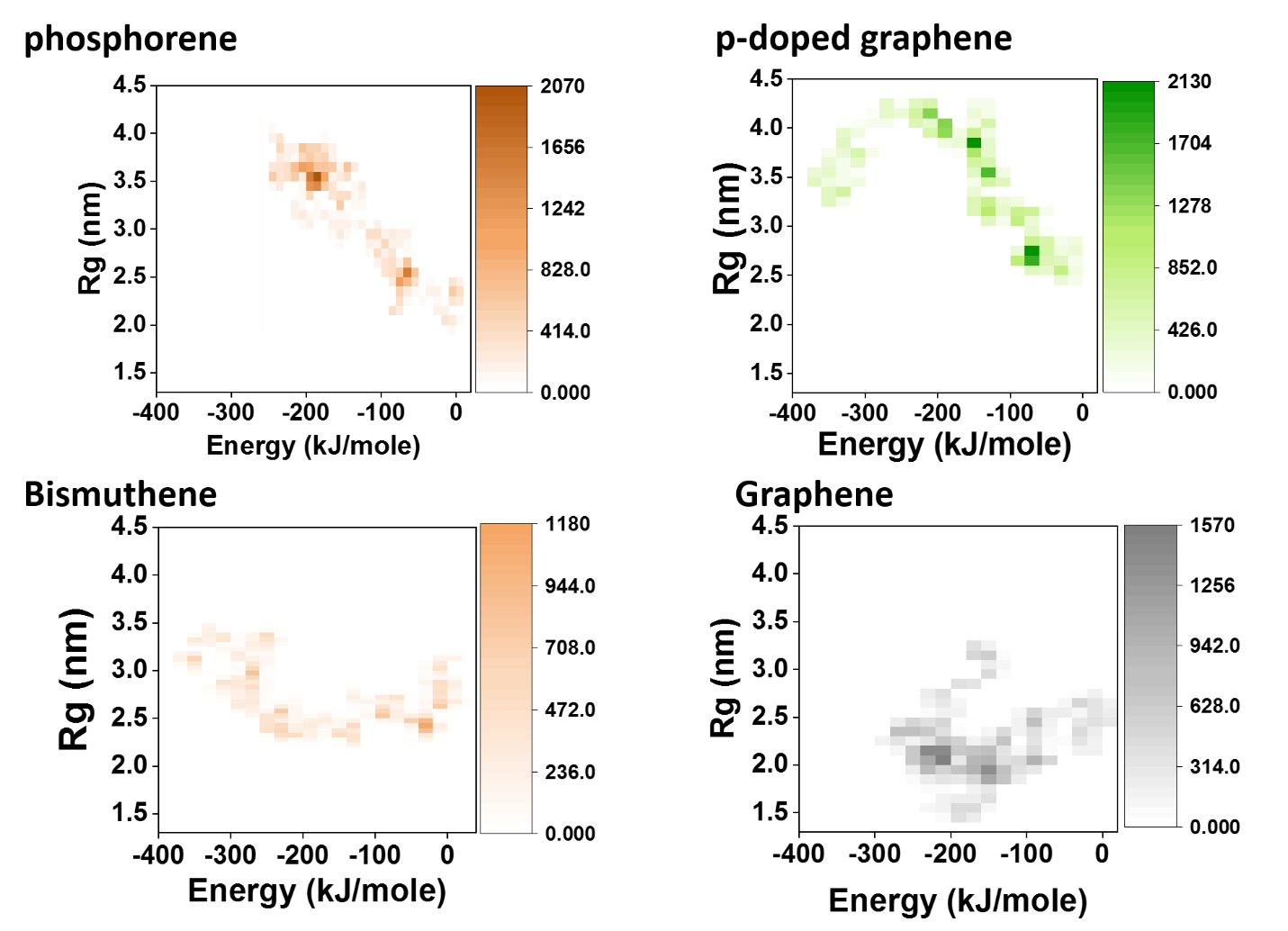


**Figure S4.** Mapping Rg vs. energy of binding for phosphorene, p-doped graphene, bismuthene, and graphene.

**Table S1.** Error bars of Energy for Nanostructures and spike protein.

| Residue | Energies | Functionalized (-) P-doped Graphene - spike protein | P-doped Graphene - spike protein | Phosphorene - spike protein | Bismuthene - spike protein | Graphene - spike protein |
| --- | --- | --- | --- | --- | --- | --- |
| THR | VDW | 0.563 | 0 | 0.286 | 0.463 | 0.908 |
|  | Electrostatics | 0.269 | 0.6 | 0.188 | 0.254 | 0.678 |
|  | Total | 0.832 | 0.6 | 0.474 | 0.717 | 1.586 |
| ASN | VDW | 0.389 | 0.3 | 0.598 | 0.407 | 0.452 |
|  | Electrostatics | 0.171 | 0.4 | 0.325 | 0.043 | 0.697 |
|  | Total | 0.56 | 0.7 | 0.923 | 0.45 | 1.149 |
| LEU | VDW | 0.566 | 0.2 | 0.746 | 0.319 | 0.615 |
|  | Electrostatics | 0.239 | 0.9 | 0.127 | 0.712 | 0.665 |
|  | Total | 0.805 | 1.1 | 0.873 | 1.031 | 1.28 |
| CYS | VDW | 0.262 | 0.3 | 0.48 | 0.249 | 0.422 |
|  | Electrostatics | 0.097 | 0.8 | 0.857 | 0.88 | 0.494 |
|  | Total | 0.359 | 1.1 | 1.337 | 1.129 | 0.916 |
| PRO | VDW | 0.864 | 0.2 | 0.309 | 0.898 | 0.61 |
|  | Electrostatics | 0.301 | 0.5 | 0.848 | 0.975 | 0.36 |
|  | Total | 1.165 | 0.7 | 1.157 | 1.873 | 0.97 |
| PHE | VDW | 0.68 | 0.2 | 0.101 | 0.1 | 0.897 |
|  | Electrostatics | 0.702 | 0.1 | 0.231 | 0.891 | 0.278 |
|  | Total | 1.382 | 0.3 | 0.332 | 0.991 | 1.175 |
| GLY | VDW | 0.03 | 0.5 | 0.694 | 0.043 | 0.717 |
|  | Electrostatics | 0.222 | 0.7 | 0.882 | 0.828 | 0.431 |
|  | Total | 0.252 | 1.1 | 1.576 | 0.871 | 1.148 |
| VAL | VDW | 0.806 | 0.3 | 0.166 | 0.965 | 0.884 |
|  | Electrostatics | 0.162 | 0.6 | 0.852 | 0.748 | 0.978 |
|  | Total | 0.968 | 0.9 | 1.018 | 1.713 | 1.862 |
| ASP | VDW | 0.216 | 0.3 | 0.516 | 0.481 | 0.479 |
|  | Electrostatics | 0.374 | 0.2 | 0.067 | 0.304 | 0.849 |
|  | Total | 0.59 | 0.6 | 0.583 | 0.785 | 1.328 |
| ALA | VDW | 0.291 | 0.8 | 0.791 | 0.316 | 0.451 |
|  | Electrostatics | 0.009 | 0.4 | 0.002 | 0.173 | 0.016 |
|  | Total | 0.3 | 1.2 | 0.793 | 0.489 | 0.467 |
| ARG | VDW | 0.336 | 0.6 | 0.471 | 0.866 | 0.208 |
|  | Electrostatics | 0.571 | 0.8 | 0.932 | 0.432 | 0.617 |
|  | Total | 0.907 | 1.4 | 1.403 | 1.298 | 0.825 |
| TYR | VDW | 0.034 | 0.9 | 0.989 | 0.374 | 0.603 |
|  | Electrostatics | 0.785 | 0.5 | 0.939 | 0.898 | 0.565 |
|  | Total | 0.819 | 1.3 | 1.928 | 1.272 | 1.168 |
| LYS | VDW | 0.655 | 0.3 | 0.593 | 0.456 | 0.445 |
|  | Electrostatics | 0.186 | 0.2 | 0.214 | 0.578 | 0.477 |
|  | Total | 0.841 | 0.5 | 0.807 | 1.034 | 0.922 |
| ILE | VDW | 0.777 | 0.6 | 0.44 | 0.694 | 0.429 |
|  | Electrostatics | 0.693 | 0.4 | 0.955 | 0.12 | 0.808 |
|  | Total | 1.47 | 1 | 1.395 | 0.814 | 1.237 |
| SER | VDW | 0.683 | 0.4 | 0.333 | 0.912 | 0.774 |
|  | Electrostatics | 0.748 | 0.2 | 0.319 | 0.476 | 0.84 |
|  | Total | 1.431 | 0.6 | 0.652 | 1.388 | 1.614 |
| CYS | VDW | 0.34 | 0.3 | 0.691 | 0.249 | 0.183 |
|  | Electrostatics | 0.405 | 1 | 0.594 | 0.598 | 0.691 |
|  | Total | 0.745 | 1.3 | 1.285 | 0.847 | 0.874 |
| GLU | VDW | 0.272 | 0.6 | 0.259 | 0.712 | 0.632 |
|  | Electrostatics | 0.733 | 0.1 | 0.021 | 0.278 | 0.357 |
|  | Total | 1.005 | 0.7 | 0.28 | 0.99 | 0.989 |
| GLN | VDW | 0.384 | 0.2 | 0.206 | 0.449 | 0.203 |
|  | Electrostatics | 0.747 | 0.3 | 0.66 | 0.552 | 0.224 |
|  | Total | 1.131 | 0.5 | 0.866 | 1.001 | 0.427 |
| HIS | VDW | 0.711 | 0.9 | 0.783 | 1 | 0.669 |
|  | Electrostatics | 0.053 | 0.4 | 0.648 | 0.251 | 0.655 |
|  | Total | 0.764 | 1.3 | 1.431 | 1.251 | 1.324 |

**References**

1. Han Y, Král P. Computational Design of ACE2-Based Peptide Inhibitors of SARS-CoV-2. ACS nano. 2020;14:5143-7.

2. Pramanik A, Gao Y, Patibandla S, Mitra D, McCandless MG, Fassero LA, Gates K, Tandon R, Ray PC. The rapid diagnosis and effective inhibition of coronavirus using spike antibody attached gold nanoparticles. Nanoscale Adv. 2021;3:1588-96.

3. Huang X, Li M, Xu Y, Zhang J, Meng X, An X, Sun L, Guo L, Shan X, Ge J, Chen J. Novel gold nanorod-based HR1 peptide inhibitor for Middle East respiratory syndrome coronavirus. ACS applied materials & interfaces. ACS Appl. Mater. Interfaces. 2019;11:19799-807.
